# When can cancer patient treatment nonadherence be considered intentional or unintentional? *A scoping review*

**DOI:** 10.1371/journal.pone.0282180

**Published:** 2023-05-03

**Authors:** Leon Wreyford, Raj Gururajan, Xujuan Zhou

**Affiliations:** University of Southern Queensland (USQ), Toowoomba, Qld, Australia; University of Sharjah, UNITED ARAB EMIRATES

## Abstract

**Background:**

Treatment nonadherence in cancer patients remains high with most interventions having had limited success. Most studies omit the multi-factorial aspects of treatment adherence and refer to medication adherence. The behaviour is rarely defined as intentional or unintentional.

**Aim:**

The aim of this Scoping Review is to increase understanding of modifiable factors in treatment nonadherence through the relationships that physicians have with their patients. This knowledge can help define when treatment nonadherence is intentional or unintentional and can assist in predicting cancer patients at risk of nonadherence and in intervention design. The scoping review provides the basis for method triangulation in two subsequent qualitative studies: 1. Sentiment analysis of online cancer support groups in relation to treatment nonadherence; 2. A qualitative validation survey to refute / or validate claims from this scoping review. Thereafter, framework development for a future (cancer patient) online peer support intervention.

**Methods:**

A Scoping Review was performed to identify peer reviewed studies that concern treatment / medication nonadherence in cancer patients—published between 2000 to 2021 (and partial 2022). The review was registered in the Prospero database CRD42020210340 and follows the PRISMA-S: an extension to the PRISMA Statement for Reporting Literature Searches in Systematic Searches. The principles of meta-ethnography are used in a synthesis of qualitative findings that preserve the context of primary data. An aim of meta-ethnography is to identify common and refuted themes across studies. This is not a mixed methods study, but due to a limited qualitativevidence base and to broaden findings, the qualitative elements (author interpretations) found within relevant quantitative studies have been included.

**Results:**

Of 7510 articles identified, 240 full texts were reviewed with 35 included. These comprise 15 qualitative and 20 quantitative studies. One major theme, that embraces 6 sub themes has emerged: **‘Physician factors can influence patient factors in treatment nonadherence’.** The six (6) subthemes are: 1. Suboptimal Communication; 2. The concept of Information differs between Patient and Physician; 3.Inadequate time. 4. The need for Treatment Concordance is vague or missing from concepts; 5. The importance of Trust in the physician / patient relationship is understated in papers; 6. Treatment concordance as a concept is rarely defined and largely missing from studies.

**Line of argument was drawn:**

**Treatment (or medication) nonadherence that is intentional or unintentional is often attributed to patient factors—with far less attention to the potential influence of physician communication factors**. The differentation between intentional or unintentional nonadherence is missing from most qualitative and quantitative studies. The holistic inter-dimensional / multi-factorial concept of ‘treatment adherence’ receives scant attention. The main focus is on medication adherence / nonadherence in the singular context. Nonadherence that is unintentional is not necessarily passive behaviour and may overlap with intentional nonadherence. The absence of treatment concordance is a barrier to treatment adherence and is rarely articulated or defined in studies.

**Conclusion:**

This review demonstrates how cancer patient treatment nonadherence is often a shared outcome. An equal focus on physican and patient factors can increase understanding of the two main types of nonadherence (intentional or unintentional). This differentation should help improve the fundamentals of intervention design.

## Introduction

The WHO assert that Interventions to improve treatment adherence could have a greater impact on population health than improvements in medical treatments [1]. However, interventions to enhance adherence appear to have only had success on a limited scale and in pilot studies. Although the phenomenon is well recognized it remains only partially understood [2]. As a result, physicians often fail to recognize nonadherence [3]. In breast cancer, medication nonadherence was shown to be a factor in almost half of patients surveyed [4]. Increased use of oral anti-cancer medications (OAM) that are self-administered and unsupervised, has increased the risk of nonadherent behaviour [5,6]. The overall rates of nonadherence for cancer patients using oral medication can vary from 16–100% [5]. In breast cancer, studies suggest that up to 50% of women discontinue or take the incorrect dosage of adjuvant endocrine therapy [7]. Nonadherence results in lost opportunities for clinical trials and increased potential for inaccurate outcomes [8] Most studies into nonadherence of oral medications have been quantitative and lack rich qualitative explanations [9]. The problem is compounded by a lack of differentiation and ambiguity between the terms ‘treatment adherence’ and ‘medication adherence’; The holistic term **‘treatment adherence’** is rarely used in the inter-related (multi-dimensional) factorial context defined by the World Health Organisation [1].

The narrow clinical term **‘medication adherence’** (and / or compliance**)** relates to medications or prescriptions and is the prime definition in studies that concern cancer patient treatment adherence or nonadherence. This may have limited the way medication or treatment nonadherence is perceived. The two concepts are used interchangeably and mostly in relation to medication adherence. There has been scant study into the multi-factorial nature of treatment nonadherence [10]. A preponderance of evidence from meta-analytics provides the building blocks to understanding nonadherence but lacks rich qualitative explanation of the cancer patient experience. In this context, a series of complex overlapping factors may be hidden from patient surveys that are used to assess nonadherence. This may be due to quantitative surveys that have been developed from a clinical needs perspective and a psychometric approach that fails to reveal the patient’s lived experience [11,12]. (Some studies promote singular solutions to a multi-faceted problem such as electronic pill counters and blood tests. However, interventions such as these resemble compliance initiatives. They may be of greater value when nonadherence is unintentional due to i.e., forgetting. Although forgetfulness may not always be a factor when patients are motivated [4]. In this context, a lack of motivation can be due to modifiable factors that may for example include, poor communication with healthcare professionals. In one study into breast cancer treatment adherence from a cohort of 181 patients, it was found that only 7.5% forgot to take their medication [13]. This result suggests that intentional nonadherence may occur more often than is measured in surveys.

It is also significant that studies into the effectiveness of patient reminder tools i.e., chronic myeloid leukaemia (CML) have had little success [14]. Therefore reminder tools may have a questionable impact as standalone solutions, although often the focus of interventions. Such as a widescale text messaging intervention in Germany to improve compliance to adjuvant endocrine therapy (AET). When the text messaging arm was compared with the control arm, there was no difference in adherence after three years of implementation [15]. A possible shortcoming of this intervention is that barriers to unintentional nonadherence may have been met—but not intentional nonadherence.

Treatment nonadherence is a multi-dimensional and multi-factorial phenomenon. However, the prime focus of this scoping is the patient dimension in relation to physician communication and associated factors. In this scoping review, although not a clinical study, there are unavoidable overlaps with Therapy and Health System dimensions due to the central role of physicians and the WHO definition of treatment adherence that includes physicians in health system dimensions.

### Nonadherence viewed as a monolithic concept

Central to this study is the lack of differentiation between intentional and nonintentional medication or treatment nonadherence. Differentiating between intentional or unintentional adherence can enable improved analysis and increased understanding of modifiable factors for intervention development [16]. A lack of attention to the differences in these concepts may be due to unintentional nonadherence being viewed as a passive behaviour and more easily identified. Decades of primarily quantitative studies and subsequent interventions to improve compliance appear to focus upon unintentional nonadherence i.e., forgetting or avoidance of side-effects and intentional. However, despite, nonadherence being viewed ‘as a monolithic concept’ the two conceptual distinctions can overlap [8,17]. (Meghani et al.,2013; Atkins and Fallowfield, 2006). As a result, there is prognostic value in maintaining an appreciation of underlying barriers that may influence unintentional nonadherence and predict future intentional nonadherence [4,18].

## The aims and objectives of this scoping review

**Fig 1:**
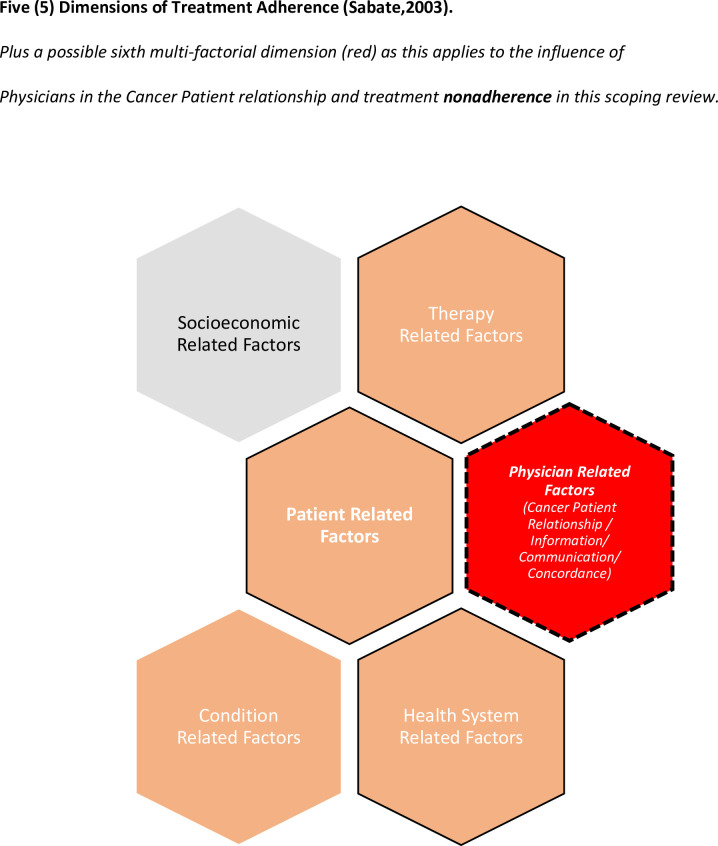
Five (5) dimensions of treatment adherence defined by WHO (2003). Plus a possible sixth multi-factorial dimension (red) as this applies to the influence of Physicians in the Cancer Patient relationship and treatment nonadherence in this scoping review.

Most studies focus on the concept of ‘medication adherence’ and use the term interchangeably with ‘treatment adherence’. Rarely do studies differentiate or combine the influence of other factors in this multi-factorial phenomenon. This scoping review explores nonadherence in the holistic context of treatment adherence as defined by the World Health Organisation in relation to chronic diseases [1]. **However, the focus of this scoping review is upon modifiable cancer patient factors and introduces ‘physician factors’ as an additional dimension in relation to communication and relationships.** Therapy related factors are often linked to physician—patient relationships and are also referred to in this study. However, unlike the multi-factorial ‘patient dimension’ a ‘physician dimension’ is not defined in the WHO multi-dimensional concept of treatment adherence. This has been included in health system related factors ref, Fig 1. This scoping review has found that information is often viewed differently by physicians and their patients. This affects patient understanding, acceptance and beliefs. When communication and relationship skills are lacking–the concept of ‘concordance’ is jeopardised. However, the term ‘treatment concordance’ receives scant mention and is often poorly defined or missing from papers that concern medication or treatment nonadherence. The concept of concordance has been disregarded in most quantitative studies due to difficulties in measurement [19].

This review finds a strong potential for physician factors to influence or overlap with patient factors in treatment nonadherence. Despite this close connection studies rarely combine this influence in treatment nonadherence. Additionally, an extensive quantitative evidence base infrequently differentiates whether treatment nonadherence is intentional or unintentional. Increased knowledge of these two distinctions can assist in the development of interventions. This study broadens knowledge of nonadherence through the identification of related themes and underlying or hidden barriers that may influence treatment nonadherence.

## Treatment and medication adherence referred to in this scoping review

**The term *treatment nonadherence* is used interchangeably with *medication nonadherence* in this scoping review. This follows the practice of authors and studies included in this scoping review. However, in the application of findings ‘treatment nonadherence’ also refers to the holistic definition of ‘treatment adherence’ provided by the WHO** [1]. In a meta-ethnographic approach, findings from a scoping review have been translated into a whole that is greater than the sum of its parts [20].

## Rationale

There has been a narrow and longstanding focus on medication adherence (ref peer reviewed articles, published circa 1995–2018 that mostly refer to medication adherence) and patient compliance (i.e., responding to the often-unquestioned advice and direction of doctors). As a result, an extensive body of quantitative literature has produced evidence concerning the extent of medication nonadherence, but with minimal rich qualitative explanation as to, why. Treatment nonadherence in the holistic sense receives scant examination. The transition from medication compliance to treatment adherence was defined by the World Health Organisation (in their reference to chronic diseases), two decades prior to this scoping review [1]. This follows a paradigm shift from the concept of medication compliance to the need for a holistic understanding of treatment adherence as a shared treatment responsibility between patient and physician [21]. This scoping review examines what impact the relationship changes have had on the experiences of cancer patients and medication in relation to treatment nonadherence.

A number of quantitative studies were found to contain significant qualitative elements (qualitative author interpretations of results) and relevance to this study. Therefore, although not a mixed study, *qualitative* summaries found in quantitative and mixed method reports are included in the scoping review *(where reference to treatment /medication adherence*, *nonadherence*, *or compliance has been made in author interpretation of results)*. These are combined with a lesser number of qualitative studies in a meta-ethnographic synthesis. Most quantitative studies have used a survey design and closed questionnaires that omit or fail to define medication / treatment non-adherence as intentional or unintentional. As a result, the limited efficacy of many interventions may be the result of an unclear characterisation of nonadherence [22]. The difficulties arise from its multi-factorial context and variables that impact the problem’s stability [22]. Increased understanding of these differences can improve the development and customisation of interventions to enhance treatment adherence [7]. The meta-ethnographic approach in this scoping review has provided a series of qualitative explanations leading to several themes, a line of argument and conclusion concerning treatment nonadherence and the relationship role of physicians. The key themes and explanations have provided rich qualitative insight into the findings of both quantitative and qualitative studies over an extensive period of over two decades.

### Future study

This scoping review provides the basis of two further studies. The research phases that follow this scoping review form method triangulation intended to validate or refute findings from this scoping review. Theses phases are: 1) sentiment analysis of online cancer support groups; 2) qualitative validation surveys of both cancer patients and treating physicians/doctors. Results from the three studies (including this scoping review) will form the basis of framework development for a future intervention to enhance treatment adherence.

### Patient surveys

Both quantitative and qualitative patient surveys often suggest that unintentional nonadherence is more prevalent than intentional nonadherence. Although this is unclear due to the sheer scale of studies that do not differentiate between the two behaviours. A common perception by patients is that deliberate nonadherence is less socially desirable, and therefore unintentional nonadherence is more frequently reported [4,8,23]. Occurrences of the two behavioural types are often inconsistent—for example, a study into adjuvant endocrine therapy found that intentional nonadherence was significantly higher than nonintentional [7].

Quantitative studies, often appear to provide results from surveys that define intentional nonadherence, such as the as negative perceptions and conflicting beliefs of patients. Less attention is given to a presumably more passive—unintentional behaviour. As a result, nonadherence is more often viewed as a patient factor and a patient responsibility [24]. An additional difficulty is discordance in how patients and health professionals report and measure nonadherence. One study has found widescale differences in patient self-reports and clinical measurements of between 10 and 20%. However, the discontinuation of medication based on urinalysis had indicated an even higher rate of nonadherence at 45% [15]. This may have been due to breaks between doses not being considered by patients as nonadherence [15] In a study concerning haematological cancers, missing doses was the most common cause of nonadherence and could not be identified as intentional or unintentional [25]. In patient self-reports changing or skipping doses is regarded as intentional nonadherence and often due to adverse effects [7]. (Brett et al., 2018). Although patients may not consider the practice of changing or skipping doses to be nonadherence. This perception may be influenced by factors such as lack of information concerning side effects, or poor communication. Patients may also overestimate adherence in their self-reports to satisfy the expectations of health professionals [26].

### Inconsistent use of terminology

The descriptive terms medication adherence and treatment adherence are used interchangeably, and often in the same context. Medication adherence refers more to ‘the process in which patients take their medications as prescribed’ [27]. Conversely, treatment adherence is multi-factorial that requires collaboration and shared responsibility [1,28,29]. In this context, there implies an understanding that the patient is not solely responsible when nonadherent [28]. The interchangeable use of terms—medication and / or treatment—is illustrated in a study by [30] in an analysis of a medication adherence self report tool ‘*Nonadherence to medication is thought to be a major impediment to achieving optimum outcomes in chronic illness*. *The measurement of patients’ adherence to treatment is*, *however*, *fraught with difficulties*. A similar example is seen in this assertion ‘*Medication adherence pertains to the degree or extent of conformity to the recommended day-to-day treatment by the health-care provider’* [31, p.59]. The dual use of treatment and medication in terminology is found throughout the literature, but rarely in the context of a treatment adherence as a multi-factorial concept. These variations appear to overlook or ignore the inter-related and multi-dimensional concept of treatment adherence that was defined and published by the World Health Organisation (WHO) two decades ago [1].

**The WHO definition of treatment adherence comprises five (5) dimensions with inter-related factors**: 1) ‘social and economic related factors’ 2) ‘therapy-related related factors’ 3) ‘condition related factors’ 4) ‘patient-related factors’ and 5) ‘health care system-related factors’ [1]. Physicians may fail to consider inter-related factors that influence treatment adherence [3,32] As previously mentioned, physician related factors are not defined in the five (5) WHO dimensions of treatment adherence but incorporated within health system related factors. However, this scoping review explores physician communication separately, and its influence in two of the five dimensions: [cancer] ‘Patient related factors’ with overlaps found in this scoping review to ‘Therapy related factors’.

### Physician factors that can influence patient factors

Patient factors that lead to treatment nonadherence are viewed separately from physician influences—although the two concepts often coexist. A direct measure of the physician’s influence is seen in a European study concerning non-persistence and treatment discontinuation in breast cancer patients [33]. This study found that clinics with a poor patient treatment compliance record have a 60% higher chance of patients discontinuing treatment than those with a good compliance record [33]. It was also reported that patients exhibit poorer compliance with GP’s than medical specialists. This was possibly due to a greater frequency of visits and stronger relationships formed with the specialists [33]. A report into patients with prostate cancer found that **‘failure of the clinical team to respond to men’s changing needs was indicated by more than two-thirds of participants as a reason for opting out’** [34, p.2733]. 1In that study, the apparent frustration was due to **‘negative experiences at diagnosis, delays and inflexibility at follow-up appointments, inaccessibility of the health care team, lack of information and support, not being able to connect with peers, being excluded from shared decision making, and perceptions of being considered a low priority were [all] reasons why men choose to discontinue despite no signs of disease progression’** [34, p.2734]. Health provider and therapy factors combined may also influence patient nonadherence due to i.e., disease progression, or intolerance of medication [35,36]. The patient may have been more tolerant to another treatment regimen and adherent [36–38].

This could then bring into question whether a patient was intentionally nonadherent. Fatigue and pain are common among cancer patients and may be undertreated [39]. Studies claim that pain is ‘experienced by 30 to 50% of patients with cancer receiving treatment, and 70 to 90% of patients with advanced disease’ [40, p.451]. Severe to moderate fatigue impacts some 30–60% of patients and can be a factor in discontinutation of treatment [5,41]. Pain management and the adverse effects of treatment are included in this review, due to their commonality in relation to nonadherent behaviour. In particular, how patients may rationalize or justify nonadherence. This may occur due to inadequate support,confusion or misunderstanding the benefits of treatment and would suggest unintentional nonadherence.

## Methods

**Fig 2:**
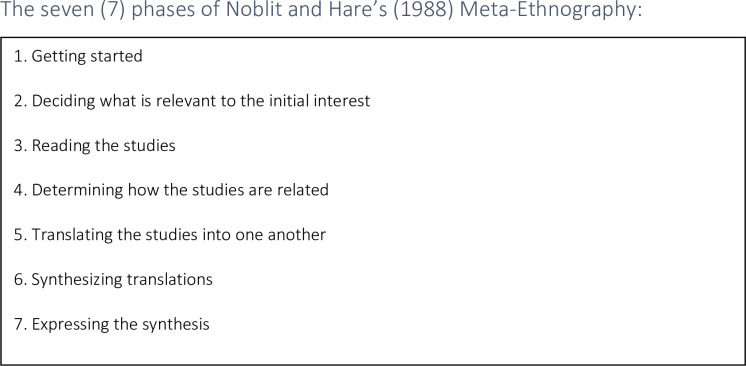
The seven (7) phases of Noblit and Hare’s (1988) [20] meta-ethnography.

The qualitative elements from quantitative studies (discussions and conclusions) that contain adequate author interpretations are included in this scoping review to broaden qualitative findings. In the context of chosen method of data synthesis … ‘Meta-Ethnography—has emerged as a potentially useful method to synthesize and integrate both qualitative and quantitative data from different perspectives using qualitative methodology’ [42,p.2]. In this research, ‘the principles of meta-ethnography are used to identify common and refuted themes across studies’ [43,p.1]. Meta-ethnography is conducted in seven phases and requires a comparison and translation of studies into each other. The collection of phases that form a meta-ethnographic synthesis are shown in Fig 2.

**Fig 3:**
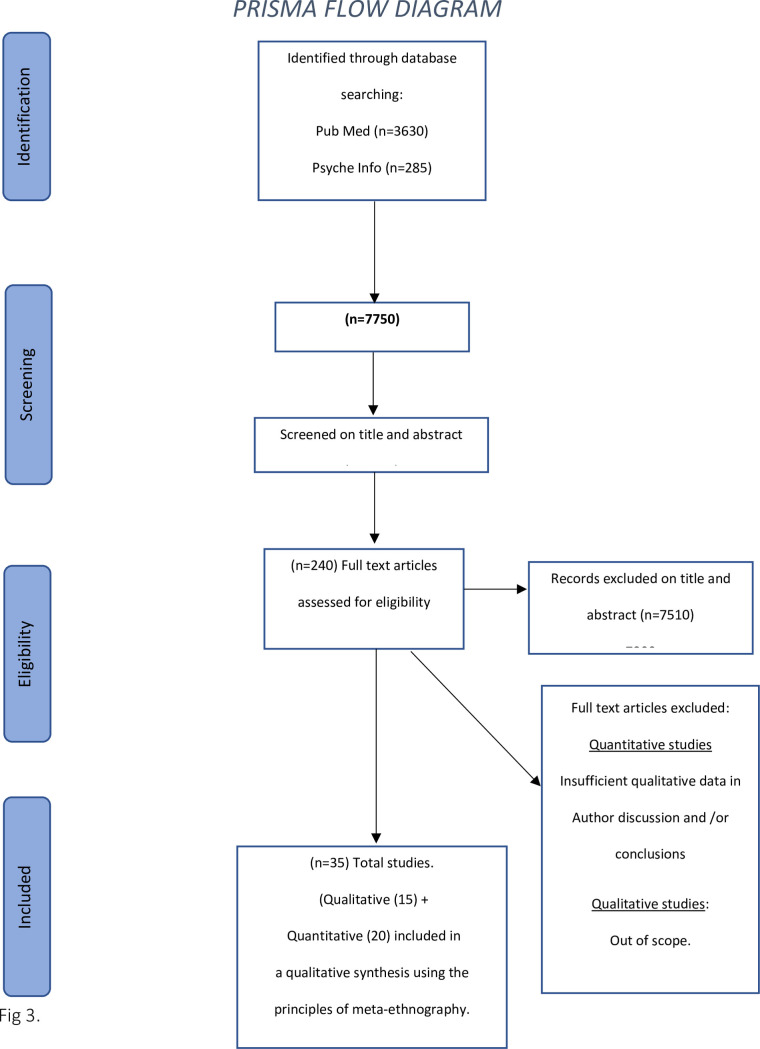
Prisma flow diagram.

A meta-analysis was not performed due to the heterogeneity of studies that concern cancer treatment, variables in study populations and differences in adherence definitions. Results are from fifteen (15) qualitative studies, plus the findings *(limited to qualitative author interpretations within discussions and conclusions)* from twenty (20) quantitative studies are synthesized as a meta-ethnography. This study has followed the Preferred Reporting Items for Systematic Reviews (PRISMA) ‘Extension for Scoping Reviews Checklist’ [44]. This includes Fig 3. The principles of meta-ethnography have been used to identify common and refuted themes across studies [20]. (Noblit and Hare, 1988).

### Characteristics

Of the studies included 15 were qualitative, plus 20 quantitative with significant qualitative elements (author opinions and interpretations of findings in discussions and conclusions). Appendix (B) refers to the characteristics of these studies. **The studies in this scoping review accumulatively represent 13,959 cancer patients and approximately 1610 treating doctors.**

The quantitative studies included are representative of an extended period (circa 1995–2015) where patient medication compliance & medication adherence studies have dominated published articles, interventions and reports. During this period there have been few far less qualitative studies into the phenomenon. Consequently, an aggregation of results would not address the research question. To extend the findings, meta-ethnography has been used in both the synthesis of qualitative reports and in author interpretations of data found in quantitative studies. This was necessary due to the scarcity of relevant qualitative studies into cancer patient treatment nonadherence. This extended approach has been adapted from other studies that have applied the principles of meta-ethnography in a similar manner [42,43,45–47]. This approach can qualitatively capture the richness of an extensive knowledge base to determine associations of quantitative evidence, and enable a more nuanced understanding [42,43,45,46]. Various supporting resources have been consulted and adapted to suit the research topic [42,43,46,48–52].

Second-order constructs from quantitative and qualitative studies are author-derived themes, discussions, conclusions, and recommendations. The extracted data has been categorised and coded according to themes. A requirement of synthesis using meta-ethnography is to preserve meanings, therefore the original text in each study has been used or paraphrased.

### Inclusion criteria

- Published peer-reviewed studies (Qualitative and *Quantitative) between 2000–2021 (to May 2022) that relate to cancer patient medication or treatment nonadherence or compliance (as seen in the title or abstract of papers).

Only *quantitative peer reviewed studies that have generated relative **qualitative evidence** in author interpretations of data, discussions and /or conclusions that relate to cancer patient treatment non-adherence.

- Reviews were excluded. The original citations in reviews were sourced for relevant literature.
- Countries filtered to: U.S, UK, Australia, Western Europe.

### Exclusion criteria

- Non peer reviewed studies.
- Cancer screening.
- Children.
- Non-English language articles, including Latin (South) America, Africa, India and China and Eastern European origin articles were filtered from search enquiries (one incidental Asian study has used in reference to opiates and pain).
- Grey literature and conference proceedings were excluded.
- The period of reference is 2000–2021. This has ensured that the WHO definition of ‘treatment adherence’ in chronic disease first published in 2003, was the approximated starting point [1]. This period defines a shift away from medication compliance—towards a shared and equitable patient and health provider relationship.

### Information sources

- Four peer reviewed publications, Pub Med, Psyche Info, Scopus and CINAHL were comprehensively searched. Peer reviewed articles that contained ‘cancer AND nonadherence’ in the title or abstract were applied. Pub Med was easily the most successful in terms of relevant papers. The search term ‘nonadherence’ was decided upon after initially searching ‘cancer AND adherence’ which returned unmanageable numbers of off-topic articles. The search was completed in May 2022.

### Quality assessment

For confidence in the evidence, studies were checked using Grade CERQual [53]. This approach is used to assess the levels of confidence that can be applied to evidence in qualitative findings and rated as: high, moderate, low, very low [53]. These grades are made ‘on the basis of an assessment of four components: methodological limitations, coherence, adequacy, and relevance’ [53]. In the final stage of the analysis, a model was developed using the principles of meta-ethnography, that makes sense of all concepts detailed in this study.

### The data collection process and data items

A Data Extraction Table 1 was prepared: First order constructs: remarks from Cancer Patients have been extracted from qualitative data such as interviews and semi structured surveys. These constructs were placed in columns under broad headings and aligned with first and second-order constructs from quantitative and qualitative data. Second order constructs from qualitative studies were contrasted with first order constructs. This would ensure that second-order constructs are grounded in the generated data [43].

**Table 1:** Data extraction.

First Order Constructs	Qualitative data: Patient’s experiences, personal remarks Physician, personal remarks. ***(shown with main text in boldened italics in between inverted single commas)***
Second Order Constructs	Qualitative & Quantitative data: Primary author interpretations of results from quantitative and qualitative discussions and conclusions. First and second order constructs combined to define conceptual categories and cluster into themes. ***(shown within maion text in bold (non-italics) in between single commas)***
Themes derived	First and second order Constructs are combined and categorised according to each study. These become third order constructs. The shared Concepts were grouped into themes and categories across studies.
Third Order Constructs	Reviewer, higher order interpretations and translations of author interpretations of cancer patient experiences concerning treatment and medication adherence / nonadherence.
Reciprocal Translation	A process of ‘putting together’ concepts grouped with similarities and refutational (with differences and in opposition to each other).
Line of Argument	Construction from third order translations to reveal the hidden meanings in each study. From these parts a whole was made concerning cancer patient treatment nonadherence in relation to the intentional or unintentional phenomenon.

## Synthesis of results

### Translation of included studies

The constructs from related headings such as information, communication and relationships were combined to form a reciprocal and refutational synthesis. These are constructs that were shared or contested and formed the synthesis and translation. Facilitating factors extracted from the quantitative papers have been categorized within the qualitative thematic framework [54]. A line of argument has then been produced. The meta-ethnographic synthesis has led to the identification of one overriding theme encompassing sub-themes. For the translation, each theme is supported by short segments of data from qualitative and quantitative studies.

1. **First-order interpretations (participants in qualitative surveys) are indicated in italics with double inverted commas. 2.Second order interpretations (descriptive accounts by authors of quantitative studies) are not italicized but shown with single inverted commas. References with page numbers follow each author or participant quotation.** This method of presentation was adapted from several similar approaches to a mixed methods review using meta-ethnography [43,45]. The various third-order interpretations are derived from the synthesis of first and second order interpretations and presented in **Table 2.** The putting together of constructs to form themes is also shown in the same table [20,43,45]. A third order interpretation is presented on the final pages of this scoping review as a line of argument, followed by a discussion and conclusion.

**Table 2:** Physician factors that may influence non-adherence.

	**Relevant Papers**	**CERQual rating**	**Explanation of Evidence**
The putting together of constructs to form themes		1.High Confidence	1.Low concerns regarding adequacy of data
**Scoping Review Findings** **1.Suboptimal communication** There is a need for individualised communication that the patient can understand. Risks and nonadherence for example can be viewed differently by patients. **2.The concept of information differs** Patients and physicans have different information needs. The information communicated to patients is relevant to clinical needs but may not satisfy patient concerns and desires i.e Quality of Life. Beliefs are formed by information, which in turn can determine adherence or intentional nonadherence. **3.Physician time** **Insufficient time with Patients impacts communication and treatment adherence.** 4.Concordance is mostly vague or missing from concepts Concordance is largely a physican factor and underpins adherence. The concept is central to patient centred care but receives scant attention. Physcians often fail to interact with patients concerning treatment adherence. **5.Cancer patient trust in the physician relationship is vital.** The source of trust may be underestimated and can be compromised.	[61]. [58] [59] [32] [60] [63] [64] [66] [11] [33] _________________ [72]. [3]. [8]. [9]. [60]. [77] [63] [36]. [34]. [4]. [36]. [74]. [71] [79] [73] [4] [58] [83] [26] [9,64]) [60]). [58] (Wright et al.,,2019) [4](Moon et al., 2017) [93] [94]. [73] [87] —**——————** [95](Guy et al.,2012) [101] [69] ______________[102] [108] [25] [106] [56] [64] [4] [78] [107] [109] [69](Arriola et al.,2014) **__________________** ——— [58](Wright et al.,2019). [56] [76] [9]. [33,78] [69] **—————**	2.High Confidence 3.Moderate Confidence 4.High Confidence 5.Low Confidence	2.Minor concerns regarding adequacyof data. 3.Minor concerns regarding limitations and adequacy 4.Moderate concerns regarding data adequacy, coherence and methodological limitaions. 5.Moderate concerns regarding data adequacy

**Appendix (A):**

Detailed Search Strategy

**Appendix (B)**

Characteristics of qualitative and quantitative studies included in the meta-ethnography.

**Appendix (C)**

Supporting thematic of Physician and Patient Factors that may influence nonadherence.

### Suboptimal communication

Health provider (in the context of this scoping review: physician) communication and information are linked to patient trust. This review has found that optimal communication is understated in studies and is not well integrated or poorly prioritised within a patient centred care approach. Patient centred care is described as having ‘**four communication domains: the patient’s perspective, the psychosocial context, shared understanding, and sharing power and responsibility**’ [55, p.1516]. The main objective of communication is ‘**to share decisional control over treatment decision making**’ and ensuring ‘**a patient’s trust in his or her physician is a key outcome**’ [56, p.591] The emphasis in studies, however, appears more toward ‘medication adherence’ (often referred to as treatment in the same context) as a patient responsibility. This shift has been led by advances in oral medication and patient preferences for the convenience this offers patients [57]. A disadvantage is that treatment is unsupervised and self-administered away from clinical settings. This sees the need for increased patient support and the need for greater communication between health providers and patients. However, poor communication, together with suboptimal patient-physician relationship are common obstacles to adherence [32,58–60] In this context, studies have found that **‘improved satisfaction with clinician communication and treatment was the most robust predictor of better adherence’** [61, p.477] In a study of communication **‘raters’**—it was found that a key reason for poor communication may be due to expectations that differ [59] This is described as a **‘divergence between physician and patient expectations of communication** ‘[59, p.5] Concerning obstacles to improving physician communication skills, a study found that **‘the greatest divergence is with those physicians who are, by external evaluation, the least skilled but the most confident in their abilities’** [59, p.2] In this study, some patients as raters, provide higher scores for communication and were less critical of their physicians—due possibly to **‘inhibition’** or **‘social desirability bias’ [**59] This report demonstrates the need for both peer evaluation and qualitative surveys, that can explore the patient’s experience to identify hidden barriers not revealed in questionnaire scores. Studies have shown that trusting relationships are formed by effective communication. One study claimed that there is a **‘highly correlated association between communication and trust, suggesting that patients who trusted their provider had a more positive experience in discussing their health concerns’** [60, p.6]. When patients experience communication clarity, greater satisfaction and quality of life is reported [62]. In this context, quality of life is a major concern of patients and contrasts with clinical benefits—a priority of health providers.

The importance of clarity is described in a breast cancer study as **‘better communication between health-care providers and patients should ultimately help to prevent refusal or discontinuation of tamoxifen treatment’** [63, p.472]. (Pellegrini et al.,2009. p.472).

In building relationships, the need for an individualised approach is of particular importance, where it was stated that **‘adherence, in fact, requires particular attention to the person, or an approach focused on providing personalized assistance, which guarantees therapeutic communication, continuity of care, direct contact with staff and the activation of functional self-care strategies’** [64, p.11]. An individualised approach that is patient centred provides the most effective approach and is a primary mediator of treatment adherence [65]. Poor health provider communication can lead to distrust and disconnection [2,26,46]. In an emphasis of the need for trust in breast cancer treatment, one author asserts that **‘for women with ovarian cancer, communication with their physician is an essential element in determining treatment course and satisfaction with care’** [66, p.148]. Agreement in goal setting and shared responsibilities [concordance] can be better achieved when communication is trustful, and the concerns of patients are openly expressed [34,60]. Even acknowledging a patient by first and second name can enhance the relationship [64] (Lacorossi et al.2019).

A caring and empathic experience is illustrated in this comment **“s*upport from the nurses has always been great*. *I don’t know how they do it*, *but they manage to say something nice to everyone*. *To show their interest*, *and even to remember people”*** [11,p.6] This study also describes the importance of empathy **‘patients were also very sensitive to the physician’s ability to listen and to reassure’** [11,p.6]. The belief that the patient is not heard was found in several studies and articulated by one patient as: … **“*And it’s also the difference between a good and a bad doctor*, *because behind that it means he’s listening”*** [11,p.6]. (Sibeoni et al.,2018, P.6).

## Information

### The concept of ‘information’ differs

Health professionals and patients have complementary aims, although clinical priorities differ from the quality-of-life concerns and expectations that patients may have. Health providers are more concerned with the disease and its symptoms rather than a patient’s meaningful life concerns [67,68]. Studies argue that **‘clinician-recorded side-effects tend to emphasize serious, life-threatening adverse events, rather than patient-reported issues affecting quality of life’** [8, p.4]. Studies claim that **‘both patients and professionals agreed on negative side effects and the information about treatment as the two main barriers and facilitators of adherence respectively, although the approaches differed between both profiles’** [9, p.1].

The difference in needs is shown in a report where **‘both patients and professionals agreed on considering the negative side effects and the information about treatment as the main barriers and facilitators of adherence, respectively, although the approaches differed between both profiles’** [9, p.10]).

A lack of information can be a major obstacle to adherence, regardless of cancer type [4,14,36]. Patients satisfied with information they receive and the impact of therapy on their Quality of Life (QOL) are more likely to be adherent [3]. (Efficace et al.,2012). A breast cancer study into adjuvant endocrine therapy adherence suggests that the patient’s experiences and beliefs shape the type of information needed. The author claims that **‘these findings suggest a very complex interplay between the physician’s medical view and advice, and the patient’s own subjective understanding of the importance of the medication, derived from her experiences’** [69, p.102]. ‘**The promotion of adherence would then require the physician to craft a rationale for taking medication that is consonant with the patient’s beliefs’** [69, p.102]. Although health professionals believe that adequate information to patients is the key to self-management of the disease, most studies report that patients receive insufficient information [9].

For example, studies have shown that only 26% of women were supported with the informational needs concerning the risk of breast cancer recurrence, and only 15% received information about the long-term side-effects [70].

Service gaps extend to education and medication lists where clinicians were found to significantly underestimate informational needs and the assistance needed [71]. This study also reported disparities in clinician practices and **‘an approach to clinical situations which may result in conflicting advice and confusion for patients’** [71, p.846].

A further study found that cancer patients like to receive positive information. This contrasted with health providers who believe that benefits and safety was of more importance. This was expressed by the author as **‘patients want more positive encouraging information from providers, but providers think patients need more information of efficacy and safety’** [72, p.125]. A separate study reported that patients did not want to hear or seek out information on side effects—as one woman puts it [not] **“*to psych myself out”*** [73, p.5] The study suggests that the emotional needs of patients are more important to them than direct discussion concerning medication adherence [73]. This assertion is supported in a report which concludes that **‘not followin prescribed regimen in cancer appears as irrational behaviour to healthcare providers, but each patient has a compelling reason for not taking medication’** [74, p.7]. There was also a misconception by physicians concerning available support. This was expressed as **‘patients appreciate support from other peer patients with similar experiences, but providers think the support from families and friends are readily available to them’** [72, p.125]. One report had found that participants in their research had **‘consistently indicated that they required information and support throughout all stages of their encounter with prostate cancer and a ‘preference …to discuss … rather than read information’** [34, p.2731].

With one patient remarking that, “***whilst there was plenty to read on the internet*, *it’s finding people that have been through it that I found most helpful” … “Often you can read and read but*, *at the end of the day*, *talking to someone*, *is the most important part”*** [34, p.2731]. Side effects are often the reason for cancer patient nonadherence in the belief that quality of life will be improved [75]. Studies into Adjuvant Endocrine Therapy (AET) claim that adequate information about side effects in advance of treatment is an important aspect of adherence. Conversely, that it may also be possible to increase nonadherence if information provided the wrong advice i.e., **‘apparently ‘helpful’ information seemed to increase non-adherence perhaps by increasing misconceptions’** [36, p.665]. Findings have shown that information is important for patients in the emotional management of their illness experiences [76]. However, health professionals **‘offered a more technical vision, while patients prioritized the emotional burden and motivation associated with the disease and medication’** [9, p.10]. In a study of chemotherapy avoidance, or potentially life-threatening delays in treatment—the need for information was highest when the number of physical symptoms were greatest—and the more **‘disgust’** a patient may feel [77, p.936]. **‘Evolutionary theorists posit that the primary purpose of ‘disgust’ is to promote avoidance of actual or potential threats to health’** [77, p.936]. The strong negative perceptions of expectations were a strong predictor of avoidance [77]. The study suggests that **‘greater disgust’** is a precursor to distress and the need for more information to reduce the risk of nonadherence [77] (Reynolds et al.,2016).

A study into tamoxifen adherence found that information should be individualized and communicated in a way that meets the needs and expectations of patients. This was due to their diverse experiences and subsequent beliefs [63]. An example of clinical facts with limited information (clinical features of treatment without patient benefits) is seen in a patient survey found in the same study;”***The doctor told me*: *‘After radiotherapy you will have a hormone*, *an anti-hormonal treatment*, *since your cancer is hormone dependent*. *It must be the same system as the pill*, *but I do not really know how it works*. *I think it blocks hormones which are targeted to the breast at certain times of the cycle*, *it puts me in a state of menopause”*** [63,p.475] The quality of information that patients receive is also of greater importance than being informed [3]. In this context, the accuracy and relevance of information should provide patients with realistic expectations. One study suggests that **‘the importance of information for adherence is associated with not having received information about side effects in advance’** [36, p.665]. A lack of information from health providers was expressed by patients in one survey in this way: **“*I think they have explained too little about side effects*. *They have minimized them*, *which makes them worse than I imagined them to be*. *Now I have to learn to deal with it after I have experienced them*, *and this is very difficult*** “[78, p.8]

***“The physician only told me I had to take the AHT medication and that I didn’t have any other choice than taking the AHT*. *She didn’t say much about side effects*. *She said that side effects were different for every person and that I would find out”*** [78, p.6] Dissatisfaction with information can result in unintentional nonadherence and negatively impact the patient-physician relationship [3].

Insufficient information or lack of clarity is illustrated in findings from a study concerning nonadherence to Aromatase Inhibitors in breast cancer survivors. It was found that **‘those who developed joint pain had a significantly lower risk of breast cancer recurrence and higher chance of overall survival compared to those who did not develop joint pain’** [79, p.7]. However, without sharing this information, joint pain severity was a strong predictor of nonadherence [79,80]. **The interpretation of information forms beliefs** Studies claim that non-adherence is related to psychosocial concepts such as beliefs, necessities and the needs of medication [18]. Beliefs are formed by the way in which information is intepreted [81] (Gleason et al.,2009) and **‘expectations influence how individuals interpret information and behavior’** [81, p.2]. (Gleason et al., 2009,p.2). However, some studies refute this, and claim that the experiences of patients and their dislikes, along with their perceptions, may be of greater relevance to nonadherence than beliefs [36]. In a similar context, one study found that perceived barriers rather than perceived benefits were predictive of nonadherence [79]. **The ‘experiences and beliefs pertaining to information was most important for perceived adherence’** [36, p.665].

This was expressed by one patient simply as **“*I expected that the pain will be gone”*** [58, p.1066]. This could place greater importance on the way in which information is structured and individualised. In a Japanese study, the question of beliefs and lack of information is seen in a patient’s comment**, “*so what*? *For example*, *to reduce the rate of metastasis by 10%*, *the agent kills normal cells as well*. *Is it good for me*? *I am concerned about it*** “[74, p.4].

### Clinical benefits versus quality of life (QOL)

In regard to medication adherence in general, studies suggest that patient concerns about the potential harms that could result from taking medications on a long term basis can result in nonadherence [7]. In this review cancer pain and the actions taken by patients to modify opiate dosage without health professional advice,was similar to intentional treatment nonadherence due to adverse effects of treatment in i.e., AHT therapy. Patients in several studies appeared to to rationalize or justify nonadherence both in cancer pain and adverse effects of medication. There is often a balance made between clinical benefits that are weighed up against side effects **‘ this weighing up process is also supported by trade-off studies showing that women with more severe side effects needed larger gains in survival to make HT [hormone therapy] worthwhile’** [4, p.27]. The side effects of Aromatase -Inhibitors, Tamoxifen side effects and opioid misuse and misunderstandings have this in common, where Patients balance the potential benefits against perceived harms [4,58,73].

In one study, this weighing, up process was described as **‘risk perception’** [73, p.10]. However, some cancer patients are found to considerably overestimate in risk perception surveys. In a report into the recurrence of breast cancer one third of patients had incorrectly doubled their estimation of risk [82]. Although the study was unrelated to treatment adherence, these findings suggest the need for greater consideration of patient risk perception in general. The authors of this report have recommended an assessment and the management of cancer patient risk, in relation to communication and **‘underlying anxiety decisions as well as communication of risk information**’ [82, p.8].

One patient demonstrates the weighing up process in her remark that **“*she was the one that said to my daughter it’s only a 20% (effective for prevention)* .. .*So*, *when I heard that*, *that’s when I said*, *‘Well*, *it’s only 20%*,*’ and I made my decision*. *Yeah*. *It’s mainly because my hands were hurting so badly”*** [73, p.10]. In managing cancer pain, patients often describe themselves as adherent when **‘skipping, lowering, or delaying’** … doses to avoid reliance on opioids [58]. **‘Despite the need to manage severe or frightening levels of pain, patients commonly perceived that the optimal approach would be to take the minimum amount of opioid needed to achieve acceptable pain control’** [58, p.1068].

Opioid Nonadherence,and variations in pain relief could also be an outcome of patient confusion [58]. One study found that safety was poorly managed and resulted in the **‘incorrect use of medication’.due to ..’lack of help seeking behaviour’**[71, p.848]. These barriers would suggest patient and health provider factors combined. There appeared to be a risk of unintentional nonadherence in almost half of the patients surveyed in the report [71]. The need to balance side effects with quality of life suggest a lack of support, particularly in the non-clinical setting and self-administered treatment. Inadequate support in self-management of oral medication is a health provider factor when identified as an obstacle to adherence.

The lack of support is illustrated in this remark**: *“It just seems to be*, *‘Right*, *you’ve had your operation*, *you’re fine cheerio now*, *we’ll see you in six months time’*, *and you’re out the door and that’s it*, *get on with your life again”*** [83,p.25]. The author of the study claims that **‘although our research demonstrates that people can successfully self-manage problems following cancer treatment, there is a need for a supportive infrastructure for survivors of cancer that takes into consideration low confidence as a significant barrier to support. Some struggle to self-manage problems without the necessary confidence, appropriate information, health care and support’** [83, p.25].

The need for self-management support may be hidden in patient surveys where nonadherence is attributed to patient factors. Deficits in knowledge clearly increase the risk of unintentional nonadherence [26,35]. However, several interventional studies question the value of increasing patient’s knowledge of treatment benefits. A large clinical trial in Germany found that an educational intervention which focused on the benefits of treatment, along with information on management of side effects—did not reveal any improvements in adherence [15]. The complexity of medication adherence is remarked by a patient who describes feeling overwhelmed and confused by a treatment regime *“****they give you the most enormous bag [of tablets]* …*they hand it to you as if*, *you know*, *now go away and you know*, *use it*”** [26, p.4]These remarks suggest that information with education and supporting activities that is needed to reduce confusion and enhance adherence, is missing [35,84]. However, the evidence that health literacy can improve adherence, remains unclear [85]. A possible consequence of poor communication and a lack of information or how it is perceived relates to adverse effects and cancer pain that patients may often experience. These concepts are defined separately in studies yet impact quality of life a chief concern of patients. The adverse effects of treatment in cancer patients is recognized as a major barrier to adherence [9,64].

An outcome is that nonadherent patients can experience lower quality of life and shorter periods before disease reoccurrence [86]. One study into the adverse effects of chemotherapy had found that patients may under-report adverse effects for fear of treatment being stopped [87]. This fear could result in unintentional nonadherence due to patient factors that are undetected. The implications of not being truthful in surveys may not be understood by patients. In this context, the author concludes **that ‘patients should be encouraged to be forthcoming about their own experiences with nausea and vomiting without fear of negative consequences (e.g., stopping treatment)’** [87, p.5].

The focus of studies into nonadherence usually includes the impact of adverse effects but much less about cancer pain. However, pain and adverse effects both rely upon treatment adherence for clinical benefits and in maintaining quality of life. In this context, patient satisfaction with pain management that is derived from effective communication, may have a stronger relationship with treatment adherence [60]. One study has found that women experience pain more than men although nonadherence is similarly high in men and women [67].In the context of severe pain **‘intentional and unintentional deviations from prescribed opioid schedules highlight the need to enhance adherence communication, education, and counselling, to optimize the use of long-acting opioids as a component of cancer pain management’** [58, p.1062].

The management of cancer pain is significant in any evaluation of cancer patient treatment adherence, as acute and chronic pain is a factor in 30% of recently diagnosed patients rising to 80% of cancer patients in an advanced stage of the disease [88]. In head and neck patients the prevalence of pain is particularly high [89]. Overall, cancer pain impacts 66% of patients across all cancer types [90,91]. The evidence suggests that management of cancer pain is far from optimal [92]. Patients describe cancer pain in this way **“*I felt so low*, *was having suicidal thoughts*, *really didn’t feel like myself at all*, *I was in so much pain and that I’d made the decision that I was going to come off tamoxifen “***[4,p.18](Moon et al.,2017, p.18).

Paradoxically, pain relief was found in one study not to be the main factor in treatment adherence [60]. Conversely, it was found that **‘satisfaction with pain treatment may depend less on symptom relief, but rather on ancillary social, health, and system level factors that influence the extent of satisfaction at the patient and provider levels’** [60, p.2]. **‘This may be particularly relevant among older adults who are more likely to have their pain mis-diagnosed and undertreated** ‘[60,p.2].

An example of suboptimal communication and confusion concerning pain, is seen in a study where it was found that joint pain and a fear of ageing could result in nonadherence [79]. There is a likelihood that patients would not realise that the treatment was effective, due to insufficient information.

The author argues that **‘sharing these research findings with patients and making patients aware of the positive associations of i.e., joint pain may help them re-conceptualize their side effects as a sign of increased longevity, as opposed to a sign of aging’** [79,p.7]

### Bother, communication and QOL

The importance that patients place on adverse effects that impact their quality of life

or **‘bother’** is claimed not to have been measured–unlike **‘SQOL’** (Symptom Quality of Life) [93]. However, knowing what is most bothersome to patients can provide many benefits such as, **‘knowledge of symptom intensity, frequency, and bother reported by patients before and during treatment would help health providers identify and address patients’ issues, integrate the information with objective clinical data, and provide required therapy modifications, supportive care, and/or self-care education’** [93, p.128]. In this context the author presents this solution **‘Why don’t we just ask our patients what is bothering them the most?’** [93, p.128].

However, it is claimed that in practice this would be difficult **when ‘communication barriers exist, and there is evidence that clinicians interrupt patients during symptom reports and use close-ended questions more often than open-ended ones, precluding a full description of the symptom. Furthermore, patients may want to be the good and strong patient, reluctant to verbalize problems’** [93, p.128].

A separate study concerning bother, argues that **‘an underestimation of patients bother may have been responsible for lower referrals for further symptom management. This in turn could negatively affect patients’ compliance with treatment of side effects because distressed patients may find it difficult to process the information they receive, leaving them vulnerable to unfavourable effects on the treatment outcomes’** [94, p.994]. It was also found that **‘the most bothersome symptom gives the clinician a place to start when synthesizing all information about a patient during cancer therapy’** [93, p.134]. In this report pain and fatigue were reported as the most bothersome. Other health related quality of life issues include concentration, appetite, and nausea [93].

### Trust and adherence

Trust in the physician relationship is an essential motivation for continued treatment [34,56,58,74]. Underlying barriers to adherence may not be recognized if the health professional and patient relationship lacks trust,and communication is poor [26]. Improving the quality of relationships and trust is vital in enhancing patient adherence. ‘**Effective communication skills among health professionals, aimed at building trust in patient-clinician relationships, providing opportunities for shared decision-making and developing self-efficacy, along with structured information and support, are key to enhancing long-term adherence’** [34, p,2728].

Trust is essential in relationships that achieve treatment concordance. In one report this need is reported as **‘relationship quality was a composite of the extent to which participants perceived that their physician shared decision making with them, the extent to which patients trusted their physician, and their closeness with their physician’** [56, p.591] Additionally, **‘the extent to which the importance of treatment goals are expressed by the healthcare provider is often contingent on the patient’s trust and confidence in issues surrounding how effective that information is communicated, along with the level of comfort the patient has in presenting concerns’** [60, p.2]. It was found that ‘**information needs, satisfaction with care and trust in the physician were seemingly interconnected’** [76, p.319]. The source of information can be a significant factor in quality and trust. A study had found that the oncologist or haematologist depending on the type of cancer was the most importance source of information [9]. In this context, the oncologist was also found to be the most frequently seen by breast cancer patients and urologists in prostate cancer [95].

The quality of patient relationships with their oncologist are among several robust psychosocial correlates of nonadherence [96]. Greater patient adherence is shown to be linked to the oncologists that prescribe patient medication [69,97]. However, patients often look to other sources in regard to further understanding their treatment.

The patient’s wider quest for information is illustrated in one study concerning endocrine therapy as **‘information from other people, online resources, and the oncologist all competed for the women’s understanding of their AI-treatment’** [73, p.7]. This suggests that patients often have difficulties in interpreting treatment information and need further assurances or explanations from others. One study had found that realistic expectations could improve adherence and would be better understood through the experiences of peers [98]. The study concluded that a physician was possibly the ideal team member to introduce a peer support intervention due to high levels of patient trust [98]. Although trust is an important feature in adherence, several studies warn of risks associated with relationships that overly emphasize trust. They allude to a fine line between trust and patient centred care that advocates shared responsibilities. This occurs when trust is prolonged, patients may become less involved in treatment decision making. This can increase the risk of unnecessary or non-preferred treatment and unsatisfactory outcomes. A study of prostate cancer patients claims that **‘following physician recommendations can have negligible or adverse clinical effects when recommended treatments lack clinical efficacy or costs outweigh benefits’** [56, p.581]. In Prostate Cancer this is due to the inherent risk of being under or over treated, and the various treatment options, plus side-effects that the patient needs to consider [56].

Other studies demonstrate the potential for harm to relationships that rely upon trust and less on shared responsibilities. Too much trust in the physician can result in an **‘unintended negative consequence’** that results from patients **who ‘may not ultimately choose the best course of treatment for themselves’** [56, p.582]. This could result in the breakdown of relationships **‘when patients thought that they had been misled as a result of poor communication, the trust in the physician was irretrievable’** [78, p.10]. The fine line in a trusting relationship demonstrates the need for patient centred care that advocates shared responsibilities and equal decision making.

### Time allocation

Patient relationships improve when the time for consultations is longer [99]. In the same context, the time pressure that physicians are often under is recognized as being a potential barrier to evidence-based care [100]. The availability of time that is allocated to patients to foster meaningful communication about their individual concerns is lacking. Studies have shown that ‘the most powerful predictor of the quality of management of chronic disease was the length of the consultation ‘[100, p.2]. In cancer, this is described in one study as **‘spending enough time with patients, listening carefully to patients’ questions and concerns particularly about treatment side effects, and explaining treatment in a manner that patients can understand, may lead to improved medication adherence’** [69, p.102].

However, the same study argues that frequency of time spend with the physician is also associated with improved adherence [69] Increased frequency of physician interaction can help overcome patient concerns about medication beliefs and enhance adherence through stressing the importance of treatment. Taking the time needed to understand patient beliefs is essential in tailoring the appropriate communication [69]. One study argues that inadequate time brings to attention a series of potential repercussions. The report claims that **‘shorter outpatient visits have been shown to be associated with decreased patient satisfaction and trust, less attention to patients’ psychosocial problems, and a decrease in the provision of certain preventive health services** ‘[95, p.2] Conversely, time spend dwelling on side effects and increasing patient concerns could be detrimental to adherence [69]. This demonstrates the strong need for communication skills that can cater for individual beliefs and levels of health literacy.

This was expressed by the authors as **‘the promotion of adherence would then require the physician to craft a rationale for taking medication that is consonant with the patient’s beliefs’** [69, p.102] In a Dutch study, insufficient time was considered by health professionals as the main barrier to both communication and providing information. However, health professionals believed that lengthening the trajectory of communication and providing patients time to communicate their questions could help resolve the problem [101].

The connection with time and the push for performance has the potential to negatively impact patient communication **‘there is a clear trade-off between physician productivity and the quality of care provided’** [95, p.2]. The study claims that **‘shorter outpatient visits have been shown to be associated with decreased patient satisfaction and trust with the aim being to meet productivity goals and thus aim to get patients in and out more quickly ‘**[95, p.2]. The detrimental impact of limited time upon patient-physician relationships is illustrated in this author sentiment **‘however, many women report difficulty having discussions about goals, priorities, and quality-of-life concerns with their oncologist, often citing insufficient consultation time’** [66, p.148] A physician sums up the lack of time for deeper patient enquiry in this manner, *‘but just the question***, *“what do you actually expect from me”*, *or “what do you think about how things will go in the coming year”*, *or*, *“will you still be here in a year”*, *or “do you expect to be there in a year”***? .. . *“****The eight-minute consultations we now have—my consultations last eight minutes on average—are not suitable for this”* .. .. *“The moment you ask such a question*, *you already know that it will take a while*. *However*, *you should actually do that”*** [101, p.9] However, other barriers **‘are the limited communication skills of HCPs themselves and their difficulties to make complicated medication-information understandable to patients, information they need to participate in decision-making’** [9,101].

Limited time illustrates the reluctance by physicians to ask potentially time-consuming open-ended questions and get to know their patients better. Improving the communication skills of physicians may stress the importance of building time into relationships to enhance treatment adherence. Increasing the frequency and quality of short consultations may assist.

## Concordance

### Concordance in physician-patient relationships appears vague

The concept of concordance underpins patient centred care—although the descriptive term receives scant reference in studies. In the healthcare setting, concordance is a mutual understanding or agreement concerning treatment between physician and patient [1,102–104]. A lack of concordance is shown to result in expectations that differed between patient and physician. One study found that **‘approximately one third of the patient population, maintained expectations about the intent of treatment that differed from those of their providers or did not comprehend the anticipated adverse effects’** [105, p.802]. Concordance has been described as a narrow version of adherence that could be applied where long-term patient physician agreements are made [28]. However, concordance is not a synonym of adherence [103]. Some studies argue that there is a difficulty in measuring concordance and that there is only an assumption that concordance leads to compliance or adherence [106,107].

This claim places some doubt on whether instances of nonadherence can be accurately defined as a patient factor when the certainty of mutual agreement with treatment (concordance) is missing. By way of example, in a comprehensive study concerning oral cancer adherence in older adults the term concordance receives no reference and is seen in only one of eighty-four citations [31]. In a report into chemotherapy treatment plans, only a relatively low number of patients were in full concordance with their physician regarding chemotherapy treatment plans [102]. This was due to a **‘suboptimal understanding of aspects of their chemotherapy treatment plans’**[102, p.11]. In that study there were anomalies, such as **‘only 57.0% of hospitalized patients reported that they understood the potential adverse effects of their medications on discharge, physicians [however] believed that 89% of their patients understood these effects’** [102, p.11]. A lack of concordance may result in patients making treatment decisions ‘**on the basis of an inaccurate understanding of prognosis or treatment-induced symptom burden may result in unrealistic expectations’** [105, 801]. The study found that a substantial number of **‘patients anticipated a more favourable outcome than did their providers’** [105, p.801]. (Duckworth et al,2022, p.801). A lack of concordance can also result from the cultural indifferences of clinicians to enter discussions and record information in medical notes that concern serious illness or end of life matters [108]. Concordance regarding the patient experience was found to be missing from notes made by physicians in almost half of all occasions [108]. This concern was expressed in one study as **‘even when clinicians did document in the EHR, only two thirds of the conversation documentation was in a structured, easily accessible source in the EHR**

**[Electronic Health Record]** ‘[108, p.9]. Other studies have confirmed similar discrepancies with one author stating that the clinical reports of their patients **‘symptoms mostly do not agree with the patients reports of their own symptoms’** [94, p.989]. A similar lack of concordance was found in a study into patient- reported outcome measures, where there was found to be **‘discordance between patient-reported symptom severity and oncologist documentation in the medical record’** [109,p.1,4]. The report provides evidence to suggest that **‘patients are better at reporting their own symptoms than physicians are at recording them’** [109, p.1,4]. n haematological cancers, one study reported that clinicians provide poor follow up support and inadequate monitoring of medication adherence [25]. In the report it was found that almost 40% of patients were never asked about medication adherence by their G.P. The study concluded that **‘clinicians are not routinely monitoring adherence’** [25, p.7,8] In contrast, a motivational intervention to engage patients and improve education was shown to improve treatment adherence [110]. Concordance (in the context of a prior agreement) may lapse if the patient does not receive follow up. This could be construed both a physician and patient factor combined and potentially result in unintentional nonadherence. Additionally, concordance can be impacted by the personal traits of physicians—despite sharing of information. This is apparent in doctors with poor relationship skills. The author of the report described the behaviour as providing **‘information to the patients without showing enough empathy or thinking about the patient’s perspective’**[99].

Studies define the relationship attributes needed for concordance as patient centred communication that is **‘skilled in informing, showing respect, and supporting patient involvement [that] can transcend issues of race and sex to establish a connection with the patient that in turn contributes to greater patient satisfaction, trust, and commitment to treatment’** [107, p.203].

### Relationships

The impact of relationships upon patient cooperation and treatment adherence can be significant as demonstrated in various studies [33,56]. Uncertain patients may require a stronger relationship to follow recommendations. For instance, ‘**when oncologists discuss clinical trials in a communication style that is informative, warm, responsive, and caring, patients are more likely to agree to join the trial ‘**[81, p.2]. One study describes communication as frequently underperforming and identified a **‘disturbed equilibrium in the doctor-patient relationship’** [33, p.2]. The patients of doctors that practice patient centred behaviour over a long period are more willing to disclose information.

This is associated with better health outcomes and reduced mortality [111]. However, patient satisfaction can decrease if the doctor does not continue to develop the relationship [111]. elationship building of this kind is seen to be a health provider factor, with less onus on the patient for non-adherent behaviour. Health providers underestimate the importance of relationships and health outcomes, there is a need for both patient feedback and specific training in this area [56]. Speaking on behalf of women, it was suggested that health providers lacked empathy with one physician claiming “***this is the one thing that I do find a lot of women struggling most with*, *that they feel so…they’re just not listened to*. *They’re not being validated in what they’re experiencing”*** [4]. This was summed up in the study as **‘some HCPs [Health Care Professionals] dismissed or belittled the side effects women were experiencing ‘** [4]. A separate study expressed the need for **‘an equal relationship where they are being taken seriously by healthcare professionals encourages them to express their difficulties and feelings’** [78, p.10 Emotional distress may have an equal or greater impact on adherence than adverse effects [88,112]. Recognizing emotional distress may reduce unintentional nonadherence, i.e., when motivation is low unintentional adherence may result [22]. Participants in surveys report **‘not feeling recognised when follow-up examinations feel rushed, when side effects are minimised or brushed aside, or when self-discovered solutions to deal with side effects are not taken seriously’** [78, p.7]. **‘Patients were told that their symptoms were not associated with tamoxifen, which left them feeling invalidated and frustrated’** [4, p.22]. This lack of validation concerning adverse effects is reflected in sentiments such as these: **“*When I mentioned side effects*, *I got the reaction*: *You have to accept you are getting older* …*At that point I disconnected myself from the conversation; there was no longer any point in talking to this person”*** [78, p.7] *…*

***“I would like to say to the physicians who work by appointment*, *to take a little more time and to listen a little better*. *I had the feeling that I could not be outside fast enough*. *I was not dressed yet and he was already writing a prescription*. *That hurts “***[78, p.7]. The risk of nonadherence increases when the patient’s perspective is not known when treatment decisions are made [103].

**Definition of first, second and third order constructs as applicable to a Meta-Ethnography** [20]. **seen on the following page is an adaptation for this review from Feast et al** [42, p.9,10].

Third Order Interpretations are derived from Reciprocal Translations.

**Please Note:** First and Second Order constructs referred to in the text are found within the various **“Relevant Papers”** listed in below table. **The quotations from qualitative papers are found in the main text–bold in between *“double commas & in italics”*. Qualitative Author interpretations from quantitative papers are seen in ‘bold, non-italics, and single commas’.**

**Line of argument synthesis:** Cancer patients have unmet needs that relate to their quality-of-life concerns that may not be satisfied by clinical benefits. Treatment adherence is influenced by the strength of health provider alliances, quality of information and effectiveness of communication. Many physicians do not routinely monitor adherence with their patients and may not fully report interactions into medical records. The beliefs that patients have may change over time, and when unknown can become a barrier to adherence. An optimal patient-physician relationship includes the timely exchange of information and performs a vital role in alleviating patient concerns and patient centred care. Failure to achieve treatment concordance increases the risk of nonadherence. Allowing insufficient time for patient interactions can be a significant barrier to concordance. The information needs of patients are often underestimated and may differ in how physicians perceive information; the interpretation and clear understanding of information that patients receive is needed to enhance adherence. The experiences of others, including peers can validate cancer patient expectations. A feature of effective patient-physician relationships is the use of empathic communication and in the provision of information that is understandable, accurate, and tailored to individual patient needs. This approach is not static and should be adapted to the current health and emotional status of patients. To maintain an optimal patient-physician alliance there is the ongoing need for support and feedback from patients. To improve the relationship and communication skills of physicians, peer assessment is also needed. Patient factors, that are attributed to intentional or unintentional nonadherence are often shared–and may at times be entirely due to modifiable physician factors.

## Discussion

This review has focused on defining patient and physician related factors in treatment nonadherence. This is considered a health system factor due to the absence of physician related factors as a separate dimension in the WHO multi-dimensional definition of treatment adherence ref Fig 1. The onus for medication / treatment adherence appears heavily weighted towards the patient and much less a shared physican responsibility. This is indicated in surveys that mostly exclude the multi-dimensional aspects of treatment adherence or the influence of physician related factors in their results. The studies included in this review show a strong correlation with ‘physican related factors’ that can influence ‘patient relation factors’ in relation to communication needs and medication / treatment nonadherence. In most quantitative studies it was seen that patients view and prioritise information differently to their clinicians. This results in a lack of comprehension and the need to inquire into other sources of information, such as the experiences of their peers. An unmet need of patients can occur when clinical benefits are not related to their quality of life expectations. The findings in this review suggest an absence of individualised information and suboptimal relationships that produce treatment discordance. It was seen that physicians spend insufficient time with their patients and consequently may have less meaningful interactions. As a result, patient-physician relationships that could enhance adherence are often less effective. To optimize relationships and concordance, patients require ongoing opportunities to provide feedback concerning their communication and information needs. In a similar context, physicians may require assessment by their peers concerning communication and relationship skills.

## BIAS and limitations

A potential bias is due to over representation of breast cancer in published reviews in comparison to all other cancer types. The selection of quotations used in the meta-ethnography in relation to patient feedback reports that are derived from qualitative accounts and author interpretations in quantitative surveys have a potential for bias. Other possible bias relates to cancer type and variables in medication and suggested barriers to treamnet/medication nonadherence. The scoping review has been perfomed using meta-ethnography to synthesise data from multiple qualitative and quantitative sources. Cancer patient treatment nonadherence is a multi-factorial phenomenon. However, the review has been limited by time and researcher constraints to focus on the role of communication and relationships. This has been in relation to physicians and patients and cancer patient treatment (or medication) adherence and nonadherence. Comparison studies or the inclusion of other chronic diseases could achieve a broader evidence base achieving greater accuracy and increased understanding of the phenomenon. This study has been limited to treatment nonadherence in cancer patients but some features of underlying physician—patient relationship barriers (non-disease related) could apply to other chronic diseases.

## Conclusion

Themes identified in this scoping review often overlap into the finer areas of communication and relationships. Overall, they strongly suggest that the importance of physician and patient related factors in relationships and treatment concordance is unclear or not defined. Consequently, treatment nonadherence may not necessarily be attributable to the patient directly but shared with—and at times due to physician factors that may result in poor relationships. Studies often emphasize patient responsibilities in treatment adherence—with less regard to physician communication and relationships. In coping and understanding their condition, patients have unmet needs that require assurances and the interpretation of clinical information from sources beyond their physician. In subsequent research the findings from this scoping review will be subject to qualitive method triangulation through validation surveys and sentiment analysis. This will provide increased knowledge and a wider evidence base that can lead to framework development for a future intervention to enhance treatment adherence.

## Supporting information

S1 File Appendix A: Detailed search strategy. (DOCX)

S2 File Appendix B: Characteristics of studies. (DOCX)

S3 File Appendix C: *Supporting thematic of Physician and Patient Factors that may influence nonadherence*. Treatment Nonadherence Factors.
(DOCX)

S4 File Bibliography (background sources not cited in main body of article). (DOCX)

S5 File PRISMA Preferred Reporting Items for Systematic reviews and Meta-Analyses extension for Scoping Reviews (PRISMA-ScR) checklist. (DOCX)
