# Supplementary material for: When can cancer patient treatment nonadherence be considered intentional or unintentional? A scoping review
Source: PLoS One. 2023 May 3;18(5):e0282180. doi: 10.1371/journal.pone.0282180 (PMC10155980; doi:10.1371/journal.pone.0282180)
Supplement: S1 File — (DOCX) [file pone.0282180.s001.docx]

**S1. Appendix A**

**Detailed search strategy.**The search terms, ‘cancer and medication adherence’, ‘cancer patient nonadherence’ ‘cancer compliance’ and ‘cancer treatment nonadherence’ were included in most search inquiries of titles and abstracts. The use of these terms provided the most ‘on topic’ results. An explicit decision was made not to define study design or cancer type as the topic receives limited journal coverage and mostly quantitative reports. The term treatment *adherence* or medication *adherence* produced minimal on topic results within unmanageable numbers. Breast cancer and adherence to tamoxifen or adjuvant hormonal therapies was the cancer type and medication seen with most frequency in search results. This is not surprising as breast cancer is the leading cancer among women. Some three quarters are oestrogen receptor positive and require hormonal therapies such as tamoxifen to block the oestrogen cells and reduce the risk of cancer recurring [4] (Moon et al,2017). At various later stages cancer type was mentioned in search filters to receive greater variation in cancer types as this applied to patient compliance, adherence, and nonadherence. Several enquiries concerning nonadherence in other chronic diseases were incidental in search results and included in this scoping review for comparison. This is due to high rates of nonadherence in other chronic diseases and the original WHO (2003) report defining adherence that has not made a distinction in disease type. In this review quantitative studies into cancer patient medication adherence were found to dominate search enquiries.

This is probably due to the overwhelming focus on *medication compliance* and not the holistic view of *treatment adherence* as per WHO (2003) definition. The titles and abstracts in peer reviewed journals (2000 -2020) were exhaustively searched with queries that included treatment (or medication) nonadherence (with / without hyphens) unintentional AND intentional medication, treatment nonadherence, treatment adherence, medication adherence.

The search was later extended to 2021, and April 2022. Titles and abstracts were searched from four electronic bibliographic databases (Pub Med, Psyche Info, Scopus and CINAHL). Where results were found to exceed 3000 from any journal, a title screening search was made to exclude irrelevant studies. PubMed - MEDLINE produced most (approximately 65%) of relevant full access results. The citations and abstracts of titles identified in the searches were downloaded into Paper Pile reference management software and duplicates removed. Full papers were then downloaded into ATLAS ti, qualitative coding software for further reading in full, and categorisation by topic and methodology (qualitative or quantitative). Followed by line-by-line coding. One qualitative Japanese study was incidental to search enquiries (oral medication and emotional experiences of patients) and has been included due to article quality and relevant/recognized authors and topics in article citations. Concordance, cancer pain management, and physician time - were searched separately, as these factors received scant reference and were largely missing from quantitative and most qualitative studies into the nonadherence phenomenon; The relevance of these potential barriers only became apparent during the latter stage of the review. The entire search was completed in May 2022.
