# Supplementary material for: When can cancer patient treatment nonadherence be considered intentional or unintentional? A scoping review
Source: PLoS One. 2023 May 3;18(5):e0282180. doi: 10.1371/journal.pone.0282180 (PMC10155980; doi:10.1371/journal.pone.0282180)
Supplement: S2 File — (DOCX) [file pone.0282180.s002.docx]

**S2. Appendix B.** Characteristics of studies
Potential Barriers to Nonadherence boldened and underlined in Main Findings.
**QUALITATIVE STUDIES**

| **Ref. No.** Atlas.ti  Qualitative, Coding Software + Author / Year of Publishing | **Participants** | **Objective** | **Sample & Setting Selection** | **Main Findings** | **Data Collection & Research Perspective. Quality Assessment.** |
| --- | --- | --- | --- | --- | --- |
| D5  (Wright et al,2019) [59] | 17 Adults with chronic cancer pain | To explore patient approaches in managing chronic cancer pain with long-acting opioids. | Adult cancer patients  in a U.S Hospital with locally advanced or metastatic cancer prescribed an opioid. | Patients would set their own parameters for use of opioids. Concern about risks and benefits. Education & counselling required. **Potential Barrier: Lack of support.** | Electronic Pill Caps &  Semi-structured interviews.  Mixed methods |
| D54 (Moon et al,2017)  [4] | 32 Breast cancer survivors | To understand women’s experiences of taking tamoxifen and to identify factors which may be associated with non-adherence. | U.K Study. Breast cancer patients prescribed Tamoxifen. | The need for medication is weighed up with costs, side effects and importance. Patients felt unsupported and needed more comprehensive education.  **Potential Barrier:** **Lack of support.** | Interviews face to face and over the telephone. |
| D69 (Verbruggue et al,2015)  [78] | 31 Breast Cancer patients | To explore the experience of breast cancer patients treated with Adjuvant Antihormonal Therapy (AHT). | University Hospital study, Belgium. | Expectations concerning treatment, social support, and recognition from health professionals, influenced nonadherence. **Potential Barrier: Poor Relationships,** | Semi-structured interviews. Grounded Theory. |
| D73 (Lacrossi et al,2019) [64] | 13 Patients Metastatic cancer | To explore adherence to oral hormone treatment in patients with metastatic prostate cancer. | National Cancer Institute of Rome, Italy. | Patients bother is underestimated. This is associated with poor QOL and distress. **Potential Barrier: Lack of empathy and poor relationship skills.** | Mixed Methods. Questionnaire and semi-structured interview. |

| D130  (Atkins and Fallowfield, ,2006) [8] | 208 Breast cancer patients | To investigate the prevalence of factors associated with non-adherence to medication amongst breast cancer patients. | UK Hospital Clinics. Routine follow up appointments. | The role of health beliefs in health locus of control regarding expectations and beliefs; **Potential Barrier:  inadequate support.** | Semi-structured interviews |
| --- | --- | --- | --- | --- | --- |
| D134 (Boons et al,2018)  [2] | 61 CML patients | To obtain insight into reasons for medication (non)adherence in chronic myeloid leukaemia (CML) and needs / wishes regarding information and communication. | Dutch CML Patients. | Motivation with timely and extensive understandable information is needed from Health Professionals. **Potential Barrier:  Poor Physician relationship with inadequate information.** | Mixed Methods. Questionnaire and semi-structured interview. |
| D146 (Pellegrini et al,2009)  [63] | 34 Breast cancer patients | The perceptions of treatment and side-effects to adherence. | Primary breast cancer patients using Tamoxifen in consultations with Oncologists. French Regional Cancer centres. | Women’s interpretation of side effects needs to be understood – better communication can prevent refusal or discontinuation. **Potential Barrier: Inadequate Physician relationships - and information conflict (clinical views vs patient understanding.** | In-depth interviews. Grounded theory |
| D165 (Yagasaki et al,2015) [74] | 14 Gastric cancer patients | To explore the experiences of patients receiving oral anticancer agents. | University Hospital in Japan. | Emotional resistance to medication outweighed rational beliefs. **Potential Barrier: Insufficient understanding or regard to patient views** **(Poor Concordance).** | Qualitative study  using semi-structured interviews. |
| D238 (Talens et al,2021)  [9] | 23 Cancer patients  18 Health professionals | Comparison of clinical and patient beliefs | University Hospital, Spain. | Lack of information, beliefs, needs and expectations. **Potential Barrier: Clinicians placing insufficient regard to emotional needs and motivation.** | Focus Groups |
| D46 (Pieters et al,2019)  [73] | 54 Older Breast Cancer survivors | How information was interpreted. | Recruited from cancer registries in California, USA. | Misunderstandings and misconceptions about treatment **Potential Barrier: Differences in how information is interpreted.** | Thematic analysis. Interviews |

| D14 (Childs et al.2019)  [87] | 20 Cancer Patients | The lived experiences of patients undergoing chemotherapy. | Mayo Clinic. U.S Study | Better patient education and antiemetic therapy needed. **Potential Barrier: Poor Physician relationship may be a contributing factor to lack of education.** | Interviews |
| --- | --- | --- | --- | --- | --- |
| D240 (Beckmann et al, 2020)  [34] | 14 Prostate cancer patients. | Why some men on active surveillance convert to active treatment. | Royal Marsden Hospital, UK. | Long term adherence requires communication skills, trust, shared decision making, increased self-efficacy, information, and support. **Potential Barrier: support, trust and concordance.** | Semi-structured interviews. |
| D245 (Geissler et al, 2017)  [14] | 2151 Chronic Myeloid Leukemia (CML)  patients. | To assess the extent of suboptimal adherence and to investigate  motivations and behavioural patterns of  adherence in a worldwide patient sample. | Data from 106 Patient Organisation in 81 countries. | Main factors to influence adherence were the quality of the doctor-patient relationship, management of side effects and the number of doses per day. Provision of information is a major factor in adherence. **Potential Barrier: Trust and relationships, concordance.** | Questionnaires.  CML Advocates network. |
| D8  (Arber et al,2015)  [26] | 64 Cancer patients | To identify patient’s knowledge of their Oral Chemotherapy medications and their adherence. | Self-report questionnaires and in-  depth interviews in outpatient clinics. UK. | Adherence behaviour is linked to knowledge, understanding and retention of information about Oral Chemotherapy.  **Potential Barrier: Lack of support and education.** | Mixed Methods. Questionnaires. Semi-structured interviews. |
| D24  (Wouters et al,2012)  [36] | 37 Breast cancer patients | Experiences and beliefs of women treated with endocrine therapy to find determinants of non-adherence. | Breast cancer patients. Leiden University Center, The Netherlands. | Women’s experiences and perceptions concerning endocrine therapy and not just common beliefs. **Potential Barrier: Physician relationship, trust and concordance.** | Online Focus Groups, Interviews. Mixed Methods |

**QUANTITATIVE STUDIES**

| **Ref. No.** Atlas.ti  Qualitative, Coding Software | **Participants** | **Objective** | **Sample & Setting Selection** | **Main Findings** | **Data Collection & Research Perspective. Quality Assessment.** |
| --- | --- | --- | --- | --- | --- |
| D117 (Brett et al,2016)  [7] | 543 Breast cancer patients. | Study to investigate factors associated with non-adherence to support women and promote adherence. | Women from Joint Aches Cohort study. UK. | Side effects and medication beliefs were main predictors of nonadherence.  **Potential Barrier: Lack of support.** | Questionnaires. |
| D18 (Efficace,2012)  [3] | 413 CML Patients | Study to investigate patient-reported personal factors associated with adherence behaviour. | Survivors from Cancer Centres in Italy | Satisfaction with information and a higher level of support are associated with adherence. **Potential Barrier: Inadequate support.** | Quantitative Questionnaires / surveys. |
| D271 (Geerse et al,2021)  [108] | 48 Clinicians & 134 patients | The objectives were to assess concordance between written documentation and recorded audiotaped conversations. | Outpatient oncology clinics. U.S Study | Key information was missing from physician records. The question of likely prognosis was avoided.  **Potential Barrier: Poor communication and record keeping.** | A secondary analysis of data in oncology of a communication quality-improvement intervention. |
| D272 (Stanton et al,2014)  [73] | 1371 Women undergoing Endocrine Therapy | Contributors to nonadherence and non-persistence with endocrine therapy in breast cancer survivors. | Online Research Registry. U.S Study | Patient oncologist relationship impacted adherence & needs and emotions. **Potential Barrier: Underestimating patient relationships.** | Online surveys |

| D2 (Brier et al,2015)  [79] | 437 Breast cancer survivors | An evaluation of barriers to AI treatment and adherence. | Breast cancer survivors from Wellness centres. U.S Study | Perceived barriers (i.e., ageing) may predict nonadherence. **Potential Barriers: Poor understanding of pain.** | Data obtained from medical charts and surveys. |
| --- | --- | --- | --- | --- | --- |
| D38 (Moon et al,2017)  [4] | 758 Breast Cancer patients | To explore the relationship between key aspects of *CSM and *TPB and both intentional and unintentional non-adherence, to facilitate the development of interventions. | Participants recruited from 27 Oncology clinics across England. Psychosocial correlates. | Attitudes and perceptions are central to understanding adherence.  **Potential Barrier: Physician relationships** **and lack of treatment concordance.** **(CSM common sense model / TPB theory of planned behaviour}* | Cross sectional survey / Questionnaires. |
| D26 (Kostev et al,2014)  [33] | 305 Gynaecological practices. 1053 Primary care practices. 6926 Breast cancer patients | To investigate, quantify, and critically discuss the effect treating physicians have on the compliance of their breast cancer patients. | German -IMS Disease Analyser Database | Communication and exchange of information is the essential concept in adherence. Experience of the Doctor and Specialization are important.  **Potential Barrier: Lack of Treatment Concordance and patient centred care.** | Multivariate Cox regression model.  Retrospective study. |
| D18  (Efficace et al,2012)  [3] | 413 CML Patients | To investigate patient-reported personal factors associated with adherence behaviour. | Cancer survivorship study. 26 Centres in Italy | Higher levels of social support and satisfaction with information improved adherence. **Potential Barrier: Lack of functional and structural support.** | Self-report Questionnaires. |
| D101 (Baker et al,2013)  [60] | 150 Cancer patients | To determine the influence of health characteristics on satisfaction with pain treatment among older adults | Cancer centre outpatients. U.S Study | Discrimination can be determinants of QOL. Education is needed for Health Professionals in pain management. **Potential Barriers: Lack of support and understanding of pain.** | Questionnaires. U.S study. |
| D112 (Gleason et al,2009)  [81] | 101 Oncology patients+114 companions. | Whether expectations for cure prior to interacting with their oncologist influence adherence | Outpatient oncology clinics at U.S National Cancer Centres | Positive expectations lead to treatment adherence. **Potential Barriers: Lack of treatment concordance and support.** | Video recorded and coded interactions between oncologist – patient – and companions. |
| D269 (Hong et al,2016)  [93] | 775 Cancer patients | To investigate patient reported symptom distress and bothersome issues among participants with cancer | Fred Hutchinson Cancer Research Center & University of Washington Cancer Consortium and the Dana-Farber Cancer Institute | The most prevalent symptoms were bothersome issues of cough, fear, worry and insomnia. The most bothersome issue was the most important.  **Potential Barrier: Poor physician relationships and lack of support.** | Secondary analysis of data from a randomized controlled trial of the Electronic Self-Report. |
| D279 (Lou et al,2022)  [99] | 187 lung cancer patients | To examine patient satisfaction and physician factors in consultations about cancer prognosis and pain. | Data from a U.S social and behavioural influences study | Low levels of Cancer patient satisfaction were associated with increased discussion of prognosis. **Potential Barrier: Treatment Lack of Concordance / Poor Relationship skills.** | Secondary analysis of data from audio transcribed.  communication. Studies need to include more variables in patient communication. |
| D256 (Orom et al,2018)  [56] | 2008 Prostate Cancer Patients | To determine whether quality of relationships influence adherence in men with clinically localized prostate cancer. | Two U.S Cancer centres | Sharing information, trust and closeness were required in relationships.  **Potential Barrier: Lack of trust, and shared responsibility /patient centred care**. | Quantitative surveys, and medical record abstraction. |
| D263 (Chandwani et al, 2017)  [94] | 2597 Cancer Patients | The objective was to describe cancer patients’ bother due to aspects of their disease experience, and explore concordance. | University Schools of medicine. U.S Study | Lack of concordance and bother is underestimated **Potential Barrier: Treatment Concordance differs between patient and provider.** | Questionnaires. |
| D273  (Duckworth et al, 2022)  [105] | 100 Cancer patients + 34 Providers | Whether patients and clinicians agreed upon the goals and adverse effects of treatment [concordance]. | U.S Cancer Center. | Lack of concordance due to poor patient education. This has resulted in inaccurate expectations. **Potential Barrier: Treatment concordance.** | Questionnaires. |
| D16  (Maleki et al, 2016)  [71] | 53 Patient Surveys Plus 40 Clinician Surveys | To identify service gaps and unmet medication management needs. | Victorian (Australia) survey of patients who had completed Radiotherapy. | Underestimation of patient needs for medication information, education and follow up **Potential Barrier: Inadequate support.** | Surveys. |
| D44  (Brier et al,2018)  [79] | 509 Breast cancer survivors | To examine whether Arthralgia predicted nonadherence to Aromatase Inhibitors. | Data extracted from medical charts concerning adherence.  U.S Study | High levels of depression and perceived ageing increased risk of nonadherence. **Potential Barrier: Misunderstanding pain/ poor treatment concordance.** | Prospective Cohort Study. |
| D56 (Nachar et al,2019)  [109] | 82 completed PROM’s / 10 Medical Oncologists | To evaluate discordance between patient self-reports / Reported Outcome Measures - and Physician reporting. | Physician Offices where a comparison of PROM with Physician reports was made after consultation. U.S Study. | Symptoms and adherence were inconsistent with PROM.  **Potential Barrier: Poor physician relationship (reporting) resulting in misunderstanding the patient.** | Patient Reported Outcome Measures compared to EMR records. |
| D70 (Arriola et al,2014)  [69] | 200 Patient self-reports | Patient beliefs mediate the relationships between physician communication. | Medical chart abstraction and patient self-reports Study | Frequent positive / educational communication promotes adherence. **Potential Barrier: Lack of concordance and relationship skills** | Cross-sectional study. |
| D289 Burt et al.,2018)  [59] | 503 Patient-Physician pairs | To examine how ‘raters’ examine patient-physician communication. | 45 Family Physicians and 13 GP clinics in England  U.K Study | Physician self-perception of communication differs from ‘raters’. **Potential Barrier: Physician’s poor understanding of communication.** | Analysis of survey data and video taped appointments. |
