# Supplementary material for: When can cancer patient treatment nonadherence be considered intentional or unintentional? A scoping review
Source: PLoS One. 2023 May 3;18(5):e0282180. doi: 10.1371/journal.pone.0282180 (PMC10155980; doi:10.1371/journal.pone.0282180)
Supplement: S3 File — Treatment Nonadherence Factors. (DOCX) [file pone.0282180.s003.docx]

**Appendix C. Treatment Nonadherence Factors**

**2.POTENTIAL BARRIERS
Unintentional nonadherence**
Forgetting - ***HPF/PF****.*Mistakes - ***PF.***
Trust and motivation ***PF.***Medication
difficulties /complexity ***PF/HPF.***
Comorbidities - ***PF.***
Knowledge deficits
***HPF.***
Depression - ***PF.***Poor pain management
***HPF/PF.***
Long duration of treatment - ***PF.***
Intolerance - ***HPF/PF.***
Not enquiring
or recognizing nonadherence ***HPF.***
Inadequate medical records - ***HPF.***Insufficient time - ***HPF.***Depression - ***HPF/PF.***Failure to recognize communication barriers -***HPF.***Too much trust/lack of shared responsibility **– *PF***Lack of social support:
(Structural / Functional) **– PF/HS**Lack of clinical support **- HPF/HS**Poor quality Concordance - **PF**

**Legend:
PF: Patient Factor
HPF: Physician Factor / Health System (HS) related i.e., structural/functional social support.**

**HPF / PF: Combination of both Factors**

**1.POTENTIAL BARRIERS
Intentional nonadherence**Negative beliefs - ***HPF/PF.***Negative perceptions - ***HPF/PF.***
Negative expectations -***HPF/PF.***
Management of adverse effects – ***HPF****.*Quality of Life ***HPF/PF****.*Nonadherence to
lifestyle ***- PF.***Lack of Physician -empathy - ***HPF.***Lack of personalized – information - ***HPF.***
Confusion - ***HPF/ PF.***
Uninformed of consequences **- *HPF****.*
Poor pain management ***HPF /PF.***Long Duration of treatment ***PF.***
Necessity/ concerns ***HPF/PF.***Failing to recognize nonadherence ***HPF.***
Distress & Bother ***HPF/PF.***
Insufficient time - ***HPF.*** *Lack of concordance* ***HPF/PF.***
Failure to recognize communication barriers -**HPF.**Trust and motivation – **HPF**Poor quality concordance -**HPF**

- **Communication skills**
- **Interpretation of information.**
- **Relationship skills, trust.**
- **Treatment concordance.**
- **Patient support in self-administered treatment.**
- **Allocation of sufficient time.**

***Potential for overlap***
