# Supplementary material for: When can cancer patient treatment nonadherence be considered intentional or unintentional? A scoping review
Source: PLoS One. 2023 May 3;18(5):e0282180. doi: 10.1371/journal.pone.0282180 (PMC10155980; doi:10.1371/journal.pone.0282180)
Supplement: S4 File — (DOCX) [file pone.0282180.s004.docx]

**Bibliography (sources consulted - not cited in-text)**

1. Adams, R.N., Mosher, C.E., Blair, C.K., Snyder, D.C., Sloane, R., and Demark-Wahnefried, W. (2015). Cancer survivors’ uptake and adherence in diet and exercise intervention trials: an integrative data analysis. Cancer *121*, 77–83. 10.1002/cncr.28978.
2. Armijo-Olivo, S., Stiles, C.R., Hagen, N.A., Biondo, P.D., Cummings, G.G. Assessment of study quality for systematic reviews: a comparison of the Cochrane Collaboration Risk of Bias Tool and the Effective Public Health Practice Project Quality Assessment tool: methodological research. J Eval Clin Pract, 2012;18(1):12-8. [PMID: 20698919](https://www.ncbi.nlm.nih.gov/pubmed/20698919) <https://www.ephpp.ca/PDF/Quality%20Assessment%20Tool_2010_2.pdf>
3. Baglia, Michelle L., I-Hsin Lin, Brenda Cartmel, Tara Sanft, Jennifer Ligibel, Dawn L. Hershman, Maura Harrigan, Leah M. Ferrucci, Fang-Yong Li, and Melinda L. Irwin. 2019. “Endocrine-Related Quality of Life in a Randomized Trial of Exercise on Aromatase Inhibitor-Induced Arthralgias in Breast Cancer Survivors.” *Cancer* 125 (13): 2262–71.and qualitative research. Eur J Cancer Care (Engl). 2017 Nov;26(6). doi: 10.1111/ecc.12647. Epub 2017 Feb 10. PMID: 28185337.
4. Bargon, Claudia A., Marilot C. T. Batenburg, Lilianne E. van Stam, Dieuwke R. Mink van der Molen, Iris E. van Dam, Femke van der Leij, Inge O. Baas, et al. 2021. “Impact of the COVID-19 Pandemic on Patient-Reported Outcomes of Breast Cancer Patients and Survivors.” *JNCI Cancer Spectrum* 5 (1): kaa104.
5. Bhanvadia, Sumeet K., Sarah P. Psutka, Madeleine L. Burg, Ronald de Wit, Haryana M. Dhillon, Bishal Gyawali, Alicia K. Morgans, et al. 2021. “Financial Toxicity Among Patients with Prostate, Bladder, and Kidney Cancer: A Systematic Review and Call to Action.” *European Urology Oncology* 4 (3): 396–404.
6. Bøhn, Synne-Kristin H., Hanne C. Lie, Kristin V. Reinertsen, Sophie D. Fosså, Hege S. Haugnes, Cecilie E. Kiserud, Jon Håvard Loge, Torbjørn Wisløff, and Lene Thorsen. 2021. “Lifestyle among Long-Term Survivors of Cancers in Young Adulthood.” *Supportive Care in Cancer: Official Journal of the Multinational Association of Supportive Care in Cancer* 29 (1): 289–300.
7. Chan, Alexandre, Xiu Hui Low, and Kevin Yi-Lwern Yap. 2012. “Assessment of the Relationship between Adherence with Antiemetic Drug Therapy and Control of Nausea and Vomiting in Breast Cancer Patients Receiving Anthracycline-Based Chemotherapy.” *Journal of Managed Care Pharmacy: JMCP* 18 (5): 385–94. C
8. han, Amy Hai Yan, Rob Horne, Matthew Hankins, and Claudia Chisari. 2020. “The Medication Adherence Report Scale: A Measurement Tool for Eliciting Patients’ Reports of Nonadherence.” *British Journal of Clinical Pharmacology* 86 (7): 1281–88
9. Buiting, Hilde M., and Gert Olthuis. 2020. “Importance of Quality-of-Life Measurement Throughout the Disease Course.” *JAMA Network Open*.Burt, Jenni, Gary Abel, Marc N. Elliott, Natasha Elmore, Jennifer Newbould, Antoinette Davey, Nadia Llanwarne, et al. 2018.
10. Cheville, Andrea L., Steven R. Alberts, Teresa A. Rummans, Jeffrey R. Basford, Maria I. Lapid, Jeff A. Sloan, Daniel V. Satele, and Matthew M. Clark. 2015. “Improving Adherence to Cancer Treatment by Addressing Quality of Life in Patients With Advanced Gastrointestinal Cancers.” *Journal of Pain and Symptom Management* 50 (3): 321–27.
11. Chwistek, M. (2017). Recent advances in understanding and managing cancer pain. F1000Res. *6*, 945. 10.12688/f1000research.10817.1.
12. Clarijs, Marloes E., Jacob Thurell, Friedrich Kühn, Carin A. Uyl-de Groot, Elham Hedayati, Maria M. Karsten, Agnes Jager, and Linetta B. Koppert. 2021. “Measuring Quality of Life Using Patient-Reported Outcomes in Real-World Metastatic Breast Cancer Patients: The Need for a Standardized Approach.” *Cancers* 13 (10). <https://doi.org/10.3390/cancers13102308>..
13. Courneya, Kerry S., Roanne J. Segal, Karen Gelmon, John R. Mackey, Christine M. Friedenreich, Yutaka Yasui, Robert D. Reid, et al. 2014. “Predictors of Adherence to Different Types and Doses of Supervised Exercise during Breast Cancer Chemotherapy.” *The International Journal of Behavioral Nutrition and Physical Activity* 11 (July): 85.
14. Cousin, Gaëtan, Marianne Schmid Mast, Debra L. Roter, and Judith A. Hall. 2012. “Concordance between Physician Communication Style and Patient Attitudes Predicts Patient Satisfaction.” *Patient Education and Counseling* 87 (2): 193–97.
15. Dawson, Jacqueline K., Tanya B. Dorff, E. Todd Schroeder, Christianne J. Lane, Mitchell E. Gross, and Christina M. Dieli-Conwright. 2018. “Impact of Resistance Training on Body Composition and Metabolic Syndrome Variables during Androgen Deprivation Therapy for Prostate Cancer: A Pilot Randomized Controlled Trial.” *BMC Cancer* 18 (1): 368.
16. Delaney,2018 “Patient-Centred Care as an Approach to Improving Health Care in Australia.” *Collegian*25 (1): 119–23. <https://www.sciencedirect.com/science/article/abs/pii/S1322769617300422>
17. Durber, Kimberley, Georgia Kb Halkett, Michelle McMullen, and Anna K. Nowak. 2021. “Measuring Financial Toxicity in Australian Cancer Patients - Validation of the COmprehensive Score for Financial Toxicity (FACT COST) Measuring Financial Toxicity in Australian Cancer Patients.” *Asia-Pacific Journal of Clinical Oncology* 17 (4): 377–87.
18. Enting RH, Oldenmenger WH, Van Gool AR, van der Rijt CC, and Sillevis Smitt PA. 2007. “The Effects of Analgesic Prescription and Patient Adherence on Pain in a Dutch Outpatient Cancer Population.” *Journal of Pain and Symptom Management* 34 (5): 523–31.
19. Focht, B.C., Lucas, A.R., Grainger, E., Simpson, C., Fairman, C.M., Thomas-Ahner, J.M., Buell, J., Monk, J.P., Mortazavi, A., and Clinton, S.K. (2018). Effects of a Group-Mediated Exercise and Dietary Intervention in the Treatment of Prostate Cancer Patients Undergoing Androgen Deprivation Therapy: Results From the IDEA-P Trial. Ann. Behav. Med. *52*, 412–428. 10.1093/abm/kax002.
20. France, E.F., Uny, I., Ring, N., Turley, R.L., Maxwell, M., Duncan, E.A.S., Jepson, R.G., Roberts, R.J., and Noyes, J. (2019). A methodological systematic review of meta-ethnography conduct to articulate the complex analytical phases. BMC Med. Res. Methodol. *19*, 35. 10.1186/s12874-019-0670-7.
21. Garside, Ruth. n.d. “Methods for Synthesizing Qualitative Evidence.” Accessed October 31, 2021. <https://ktdrr.org/training/workshops/qual/session3/session3_031815.pdf>.
22. Hsieh, Kun-Pin, Li-Chia Chen, Kwok-Leung Cheung, Chao-Sung Chang, and Yi-Hsin Yang. 2014. “Interruption and Non-Adherence to Long-Term Adjuvant Hormone Therapy Is Associated with Adverse Survival Outcome of Breast Cancer Women--an Asian Population-Based Study.” *PloS One* 9 (2): e87027.
23. Gordon, L. G., S. M. Walker, M. C. Mervin, A. Lowe, D. P. Smith, R. A. Gardiner, and S. K. Chambers. 2017. “Financial Toxicity: A Potential Side Effect of Prostate Cancer Treatment among Australian Men.” *European Journal of Cancer Care* 26 (1). <https://doi.org/10.1111/ecc.12392>.
24. Hugtenburg, Jacqueline G., Lonneke Timmers, Petra Jm Elders, Marcia Vervloet, and Liset van Dijk. 2013. “Definitions, Variants, and Causes of Nonadherence with Medication: A Challenge for Tailored Interventions.” *Patient Preference and Adherence* 7 (July): 675–82.
25. Jacqueline G. Hugtenburg. 2018. “Needs for Information and Reasons for (non)adherence in Chronic Myeloid Leukaemia: Be Aware of Social Activities Disturbing Daily Routines.” *European Journal of Haematology*, July. <https://doi.org/10.1111/ejh.13155>.
26. Improvement: Results of a Patient-Driven Survey of 2546 Patients in 63 Countries.” *Journal of Cancer Research and Clinical Oncology* 143 (7): 1167–76.
27. Hawker, Sheila, Sheila Payne, Christine Kerr, Michael Hardey, and Jackie Powell. 2002. “Appraising the Evidence: Reviewing Disparate Data Systematically.” *Qualitati ve Health Research* 12 (9): 1284–99.
28. Henry, N. Lynn, Faouzi Azzouz, Zereunesay Desta, Lang Li, Anne T. Nguyen, Suzanne Lemler, Jill Hayden, et al. 2012. “Predictors of Aromatase Inhibitor Discontinuation as a Result of Treatment-Emergent Symptoms in Early-Stage Breast Cancer.” *Journal of Clinical Oncology: Official Journal of the American Society of Clinical Oncology* 30 (9): 936–42.
29. Hershman, Dawn L., Lawrence H. Kushi, Grace Clarke Hillyer, Ellie Coromilas, Donna Buono, Lois Lamerato, Dana H. Bovbjerg, et al. 2016. “Psychosocial Factors Related to Non-Persistence with Adjuvant Endocrine Therapy among Women with Breast Cancer: The Breast Cancer Quality of Care Study (BQUAL).” *Breast Cancer Research and Treatment* 157 (1): 133–43.
30. Howell, Doris, Alison Richardson, Carl May, Lynn Calman, Rouhi Fazelzad, Saeed Moradian, and Claire Foster. 2019. “Implementation of Self-Management Support in Cancer Care and Normalization into Routine Practice: A Systematic Scoping Literature Review Protocol.” *Systematic Reviews* 8 (1): 37.
31. Knight, Thomas G., Allison M. Deal, Stacie B. Dusetzina, Hyman B. Muss, Seul Ki Choi, Jeannette T. Bensen, and Grant R. Williams. 2018. “Financial Toxicity in Adults With Cancer: Adverse Outcomes and Noncompliance.” *Journal of Oncology Practice / American Society of Clinical Oncology*, October, JOP1800120.
32. Karimi, M., Brazier, J. Health, Health-Related Quality of Life, and Quality of Life: What is the Difference?. *PharmacoEconomics* **34,**645–649 (2016). <https://doi.org/10.1007/s40273-016-0389-9>
33. Krebs, Paul, Elliot J. Coups, Marc B. Feinstein, Jack E. Burkhalter, Richard M. Steingart, Amy Logue, Bernard J. Park, and Jamie S. Ostroff. 2012. “Health Behaviors of Early-Stage Non-Small Cell Lung Cancer Survivors.” *Journal of Cancer Survivorship: Research and Practice* 6 (1): 37–44.
34. Larena, Natalia C., Samantha L. Estevez, Susan L. Tucker, and Jacqueline S. Jeruss. 2015. “Impact of Fertility Concerns on Tamoxifen Initiation and Persistence.” *Journal of the National Cancer Institute* 107 (10). <https://doi.org/10.1093/jnci/djv202>.
35. Lehane, Elaine, and Geraldine McCarthy. 2007. “Intentional and Unintentional Medication Non-Adherence: A Comprehensive Framework for Clinical Research and Practice? A Discussion Paper.” *International Journal of Nursing Studies* 44 (8): 1468–77.
36. Lewin, Simon, Claire Glenton, Heather Munthe-Kaas, and Arash Rashidian. 2015. “Table 3. The CERQual approach—Definitions of Levels of Confidence in a.” ResearchGate. October 27, 2015. <https://www.researchgate.net/figure/The-CERQual-approach-Definitions-of-levels-of-confidence-in-a-review-finding_fig2_283294286>
37. Lewin S, Glenton C, Munthe-Kaas H, Carlsen B, Colvin CJ, Gülmezoglu M, et al. (2015) Using Qualitative Evidence in Decision Making for Health and Social Interventions: An Approach to Assess Confidence in Findings from Qualitative Evidence Syntheses (GRADE-CERQual). PLoS Med 12(10): e1001895. <https://doi.org/10.1371/journal.pmed.1001895>
38. Lu, Z. Kevin, Xiaomo Xiong, Jacob Brown, Ashley Horras, Jing Yuan, and Minghui Li. 2021. “Impact of Cost-Related Medication Nonadherence on Economic Burdens, Productivity Loss, and Functional Abilities: Management of Cancer Survivors in Medicare.” *Frontiers in Pharmacology* 12 (June): 706289.
39. Martin, Leslie R., Cheyenne Feig, Chloe R. Maksoudian, Kenrick Wysong, and Kate Faasse. 2018. “A Perspective on Nonadherence to Drug Therapy: Psychological Barriers and Strategies to Overcome Nonadherence.” *Patient Preference and Adherence* 12 (August): 1527–35.
40. Moher, David, Alessandro Liberati, Jennifer Tetzlaff, Douglas G. Altman, and PRISMA Group. 2009. “Preferred Reporting Items for Systematic Reviews and Meta-Analyses: The PRISMA Statement.” *PLoS Medicine* 6 (7): e1000097.
41. Midão, L., Giardini, A., Menditto, E., and Costa, E. (2018). Adherence to Medication in Older Adults as a Way to Improve Health Outcomes and Reduce Healthcare System. In Gerontology (unknown). 10.5772/intechopen.72070.
42. Montagna, Emilia, Paola Zagami, Marianna Masiero, Ketti Mazzocco, Gabriella Pravettoni, and Elisabetta Munzone. 2021. “Assessing Predictors of Tamoxifen Nonadherence in Patients with Early Breast Cancer.” *Patient Preference and Adherence* 15 (September): 2051–61.
43. Pagès-Puigdemont, Neus, Maria Antònia Mangues, Montserrat Masip, Giovanna Gabriele, Laura Fernández-Maldonado, Sergi Blancafort, and Laura Tuneu. 2016. “Patients’ Perspective of Medication Adherence in Chronic Conditions: A Qualitative Study.” *Advances in Therapy* 33 (10): 1740–54.
44. Post, Marcel W. M. 2014. “Definitions of Quality of Life: What Has Happened and How to Move on.” *Topics in Spinal Cord Injury Rehabilitation* 20 (3): 167–80.
45. “Principles of Cancer Pain Management.” n.d. <https://www1.racgp.org.au/ajgp/2018/november/principles-of-cancer-pain-management>.
46. Redelico, Tyler J., Suzanne M. Walton, Jennifer LaFollette, Marjorie Adams Curry, and Leon Bernal-Mizrachi. 2018. “Assessment of Provider Adherence to Recommended Monitoring Parameters for Oral Anticancer Medications.” *Journal of Oncology Practice / American Society of Clinical Oncology* 14 (7): e446–50.
47. Salsman, John M., Kristin Bingen, Ronald D. Barr, and David R. Freyer. 2019. “Understanding, Measuring, and Addressing the Financial Impact of Cancer on Adolescents and Young Adults.” *Pediatric Blood & Cancer* 66 (7): e27660.
48. Spoelstra, Sandra L., and Cynthia N. Rittenberg. 2015. “Assessment and Measurement of Medication Adherence: Oral Agents for Cancer.” *Clinical Journal of Oncology Nursing* 19 (3 Suppl): 47–52
49. Schlesinger, Sabrina, Jessica Walter, Jochen Hampe, Witigo von Schönfels, Sebastian Hinz, Thomas Küchler, Gunnar Jacobs, Clemens Schafmayer, and Ute Nöthlings. 2014. “Lifestyle Factors and Health-Related Quality of Life in Colorectal Cancer Survivors.” *Cancer Causes & Control: CCC* 25 (1): 99–110.
50. Sun, V., D. J. Raz, J. Y. Kim, L. Melstrom, S. Hite, G. Varatkar, and Y. Fong. 2020. “Barriers and Facilitators of Adherence to a Perioperative Physical Activity Intervention f or Older Adults with Cancer and Their Family Caregivers.” *Journal of Geriatric Oncology* 11 (2): 256–62.
51. Swankhuisen, Christine, Jelle Evers, L. A. L. M. Kiemeney, Nicoline Hoogerbrugge, and Femmie de Vegt. 2019. “[The relationship between lifestyle and cancer; topic of conversation in the consultation room?].” *Nederlands tijdschrift voor geneeskunde* 163 (May). <https://www.ncbi.nlm.nih.gov/pubmed/31187964>.
52. Thomas, B. H., D. Ciliska, M. Dobbins, and S. Micucci. 2004. “A Process for Systematically Reviewing the Literature: Providing the Research Evidence for Public Health Nursing Interventions.” *Worldviews on Evidence-Based Nursing / Sigma Theta Tau International, Honor Society of Nursing* 1 (3): 176–84.
53. Winger, Joseph G., Catherine E. Mosher, Kevin L. Rand, Miriam C. Morey, Denise C. Snyder, and Wendy Demark-Wahnefried. 2014. “Diet and Exercise Intervention Adherence and Health-Related Outcomes among Older Long-Term Breast, Prostate, and Colorectal Cancer Survivors.” *Annals of Behavioral Medicine: A Publication of the Society of Behavioral Medicine* 48 (2): 235–45.
